# Cost-utility analysis of integrated therapist and online CBT over and above usual care for patients with depression in primary care: an economic evaluation of the INTERACT randomised controlled trial

**DOI:** 10.1136/bmjment-2026-302884

**Published:** 2026-10-22

**Authors:** Jinshuo Li, Steve Parrott, Stephanie MacNeill, Debbie Tallon, Laura Thomas, Sally Brabyn, Brian C F Ching, Jane Sungmin Hahn, Berry Jude, Mekeda X Logan, Alex Burrage, Simon Gilbody, Paul Lanham, Glyn Lewis, Irwin Nazareth, Tim J Peters, Roz Shafran, Katrina Turner, Christopher Williams, David Kessler, Nicola Wiles

**Affiliations:** 1 Department of Health Sciences, University of York, York, UK; 2 Department of Population Health Sciences, Bristol Medical School, University of Bristol, Bristol, UK; 3 Division of Psychiatry, University College London, London, UK; 4 Hull York Medical School, York, UK; 5 Patient and Public Involvement Representative, London, UK; 6 Department of Primary Care and Population Health, University College London, London, UK; 7 Bristol Dental School, University of Bristol, Bristol, UK; 8 Great Ormond Street Hospital for Children NHS Foundation Trust, London, UK; 9 School of Health and Wellbeing, University of Glasgow, Glasgow, UK

**Keywords:** Depressive Disorder, Behavior Therapy, Mental Health Services, Internet-Based Intervention, Cost-Effectiveness Analysis

## Abstract

**Background:**

We conducted a pragmatic, parallel two-group, multicentre randomised controlled trial to assess an online platform that integrates the use of online cognitive behavioural therapy (CBT) materials with therapist-led sessions via instant messaging and found it clinically effective, over and above usual care.

**Objective:**

To investigate cost-effectiveness of integrated CBT over and above usual care over 12 months for primary care patients with depression.

**Methods:**

We undertook incremental cost-utility analyses alongside the trial. The cost components in the primary analysis were integrated CBT and usual care, and wider healthcare and social services for mental health from a National Health Service (NHS) and personal social services (PSS) perspective. The secondary analyses included costs from other perspectives. The effect was measured by quality-adjusted life years (QALYs), derived from EuroQoL 5-Domain with 5-Level questionnaire. A series of sensitivity analyses was performed to assess uncertainties.

**Findings:**

The mean costs of integrated CBT intervention were £987 (SE £14) per participant. The incremental cost-effectiveness ratio from an NHS/PSS perspective was £30 576 per QALY gained (probability of cost-effectiveness from £25 000 to £35 000/QALY: 26% to 50%). Secondary analyses also reported higher costs in the integrated CBT group from other perspectives.

**Conclusions:**

Under National Institute for Health and Care Excellence thresholds, integrated CBT is cost-effective over and above usual care over 12 months with high decision uncertainty.

**Clinical implications:**

Although integrated CBT was found clinically effective, the economic evaluation alongside the trial did not find evidence with certainty to support its implementation over and above usual care. It should not be adopted as a standalone treatment option unless long-term cost-effectiveness is established.

**WHAT IS ALREADY KNOWN ON THIS TOPIC**

- Therapist-delivered online cognitive behavioural therapy (CBT) is clinically effective in treating depression and is acceptable.

**WHAT THIS STUDY ADDS**

- This study adds to the evidence base of economic evaluations of therapist-delivered online CBT.

**HOW THIS STUDY MIGHT AFFECT RESEARCH, PRACTICE OR POLICY**

- Integrating live therapy sessions via instant messaging with access to digital CBT materials through an online therapy platform might appeal to individuals who are hesitant to engage in in-person therapy or find unsupported technology-driven approaches, like computerised CBT, unappealing. This population can fall into a gap of typically poor provision. Further research is needed to test whether access to care is improved by this provision and whether the added synchronous engagement is worth additional healthcare costs. Furthermore, a long-term assessment of this approach will provide a more comprehensive picture of costs and benefits.

## Background

 In 2024/2025, the National Health Service (NHS) Talking Therapies programme (formerly Improving Access to Psychological Therapies) reported £861 million in costs of care contact.^1^ As the main provider of cognitive behavioural therapy (CBT) in England, one in three referrals were for depression.^2^ Expedited by the COVID-19 pandemic, it is now offering several modes of treatment delivery, including in-person or remote therapy (by video call, telephone or secure messaging) to suit the patient’s needs.^3^

Digital tools of therapy delivery offer accessible alternatives that make use of the advancing technology and overcome difficulties in access, including but not limited to mobility, time and geographical constraints. With the rapid development of artificial intelligence and expansion of mobile networks, the integration of CBT and digital technologies fosters the potential for better adherence to treatment, but issues such as patient engagement, personalisation of treatment, therapeutic alliance in a digital environment and smooth integration into existing health services system remain to be addressed.^4^ Facing the demands of post-pandemic reality and people’s adaptation to online services, digital therapy delivery modes are quickly adopted, some of which lack solid evidence on clinical effectiveness, let alone cost-effectiveness. We designed and developed an online platform that integrates CBT materials and an instant messaging system, allowing high-intensity individual CBT to be delivered via live text chat and facilitated by materials that could be used within and between sessions. A 12-month randomised controlled trial (RCT) was then conducted to examine the clinical and cost-effectiveness of the integrated CBT over and above usual care.^5^

The primary endpoint of the trial was 6 months post-randomisation. The integrated CBT approach was found clinically effective^6^ and acceptable to patients and therapists.^7^ The current article presents the economic evaluation undertaken alongside the trial.

### Objective

The objective was to examine the cost-effectiveness, over 12 months, of an integrated approach to delivering CBT (which integrated therapist-led instant messaging therapy sessions with access to CBT materials through an online therapy platform) over and above usual general practitioner (GP) care for primary care patients with depression.

## Methods

### Design and setting

The INTERACT trial was a pragmatic, two parallel-group, multicentre, individual-level RCT that recruited from 67 general practices in areas surrounding the three sites (Bristol, London and York). The trial was prospectively registered in the ISRCTN registry (ISRCTN13112900). From 29 December 2020 to 27 April 2023, a total of 451 participants were randomised to either the integrated CBT group (n=225) or the usual care group (n=226). The integrated CBT sessions were delivered between 21 January 2021 and 28 September 2023. Except for 14 participants who never had any sessions and one who only had the first session, the duration of integrated CBT was on average 89.2 days per participant (SD 44.4, range 6–267, n=210). While the primary endpoint was 6 months post-randomisation, the economic evaluation took the 12-month time horizon to better capture the impact on healthcare resource use and quality of life after the intensive treatment delivery period.

Eligible participants were 18 years old or older, scoring ≥14 on BDI-II^8^ and meeting ICD-10 criteria for depression, assessed using the Clinical Interview Schedule–Revised.^9^ Those who were diagnosed with alcohol or substance dependency in the past year, bipolar disorder, schizophrenia, psychosis or dementia, or were currently under psychiatric care for depression, could not complete questionnaires unaided or would require an interpreter, were currently receiving CBT or other psychotherapy, had received high-intensity CBT in the past 4 years, were taking part in another intervention trial, or were not willing or able to receive CBT via computer/laptop/smartphone, were excluded. The published protocol provides full details of the trial settings and procedures.^5^ Findings in terms of clinical effectiveness have been reported separately.^6^

### Intervention and usual care

Integrated CBT comprised nine therapist-led sessions, with up to three additional sessions if clinically appropriate, supported by an online platform. Therapists (NHS Band 7) received training during their initial month of employment, followed by weekly group supervision sessions with an experienced therapist (NHS Band 8a) by site. The first therapy session was a 90-minute video call and the rest were 50-minute sessions via secure online instant messaging on the INTERACT therapy platform. The platform also included a collaborative workspace containing CBT resources (worksheets, psychoeducational information sheets and short videos) for both therapists and participants to use.^5^

Usual care comprised psychological services, including packages of computerised CBT (cCBT), counselling or talking therapy provided by the NHS, and prescriptions of antidepressant medicine. Both groups received usual care.

### Resources use

#### Data collection

Integrated CBT components included therapist training, weekly supervision, therapy sessions and operation of the INTERACT online therapy platform (basic maintenance, operating system end-of-life mitigations, development framework upgrade and user interface upgrades). The staff training timetable, attendance and costs of materials, venues and refreshments were recorded by the research team where applicable.

Primary care included in-person or telephone consultations with general practice-based health professionals. Information on prescribed antidepressants and primary care consultations were extracted (where consent was given) from participants’ GP records at the end of 12-month follow-up, covering the previous 12 months. Without accessing the contents of the consultations, those that were specific to mental health could not be separated from others. Hence, costs of primary care were not limited to mental health.

Other healthcare consultations (in hospital or in community) and social services included mental health-related help from a mental health nurse or community psychiatric nurse, psychiatrist, social worker, clinical psychologist and NHS- or personal social services (PSS)-funded home care, day centre and self-help group. Secondary care included utilisation of accident and emergency, overnight stay and day case for mental health problems.

Participants’ out-of-pocket payments (OOPs) included those on private therapies or alternative treatments, home care, private hospital or clinic, over-the-counter remedies and travelling to GP appointments/treatments. Lost income due to mental health included both participants’ and informal carers’.

Benefits included healthcare and social services (eg, cCBT, counselling, psychotherapy) provided/paid for by employers or voluntary organisations, paid leave (with or without temporary cover) from work for mental health reasons, exemption of prescription charge for prescribed antidepressants and carer’s allowance. These were benefits to the participants but considered as costs to society.

Data regarding the use of usual care psychological services, other healthcare and social services, OOPs and benefits were collected via self-reported Case Report Form (CRF) at 6- and 12-month post-randomisation.

#### Data valuation

All monetary outcomes were presented in pounds sterling 2022/2023, with data not available in 2022/2023 prices inflated using the Consumer Price Index.^10^

Staff costs of integrated CBT were estimated by multiplying the hours they spent by their respective hourly costs. Hourly costs included salary, oncosts and overheads and capital proportional to salary to reflect costs of internet, digital equipment, office space, etc. The duration of time of research-related training activities was excluded. The duration of group supervision was estimated to be 1 hour per week with two therapists and 1.25 hours per week with three therapists. The exact duration of therapy sessions was not recorded, so their costs were estimated based on the delivery plan. Sessions recorded as ‘did not attend/rescheduling/cancellation’ were assumed to take 5 minutes for administration.^11^ Costs of operating and maintaining the INTERACT platform were estimated based on information provided by the University of Bristol Research IT team, where it was hosted during the study.

Costs of usual care, healthcare and social services were estimated by applying a set of national average unit costs of the services, compiled from published secondary sources of the year 2022/2023 (online supplemental material 1), to the quantities collected. Participants’ OOPs and lost income were collected in monetary terms. A prescription charge of £9.35 per item^12^ was applied where not exempted. Informal carers’ lost income was estimated by multiplying the time off work by the average wages of their respective occupation categories (online supplemental material 1).

Healthcare and social services that were considered as benefits were costed using NHS costs of similar services. Those who were in full-time or part-time employment were asked if they had taken leave from work and if they had lost income because of it. If they had taken leave but not lost any income, the leave was considered paid leave. The average income of full-time and part-time employment was applied to their days of leave to estimate the benefits in terms of paid leave (online supplemental material 1). The benefits of paid leave were doubled if additional personnel were temporarily hired to cover for them. Exemption of prescription charge was estimated using identified prescribed items from general practice records and the £9.65 per item charge.^12^ The published government informal carer’s allowance in 2023 (£76.75 per week) was used to estimate this benefit where it was reported.^13^

### Effectiveness

The effectiveness measure was quality-adjusted life years (QALYs), derived from EuroQoL 5-Domain with 5-Level (EQ-5D-5L) questionnaire.^14^ It was administered at baseline, 6- and 12-month post-randomisation. Following the recommendation by NICE,^15^ the mapping approach (mapping the 5L descriptive system data onto the 3L value set) was used to execute the conversion from domains to utilities.^16^ Using the area under the curve approach,^17^ utilities at the three time points were used to derive QALYs.

### Missing data

Missing values at baseline were imputed by the mean of the measure of the pooled sample of both groups.^18^ Missing data on measures at follow-ups were handled using multiple imputation with the chained equation method by allocation, following Rubin’s rule and assuming missing at random (MAR).^19 20^ The imputation model included BDI-II tertile group, age, gender, study centre, antidepressant costs, history of depression, prior referral to psychiatrist for depression, employment status and EQ-5D-5L utility at baseline. It further included costs of integrated CBT, usual care, of categories of healthcare and social services for mental health, OOPs and lost income, and benefits at follow-ups. The costs were imputed on a category level (eg, GP-based mental health services, antidepressants, hospital services for mental health, etc). Predictive mean matching was used for imputation with 10 closest neighbours to draw from. The number of imputed datasets was set as approximately the highest percentage figure of the missing data level.^18^ Unless otherwise specified, the analyses were performed using the imputed dataset.

### Analysis

All analyses were carried out comparing groups as randomised, following a pre-specified analysis plan (https://research-information.bris.ac.uk/ws/portalfiles/portal/397633247/INTERACT_RCT_HEAP_v1.0_20240104_signed_-Signatures_redacted.pdf). Discounting was not undertaken as the follow-up period did not exceed 1 year.

The primary analysis was an incremental cost-utility analysis of integrated CBT over and above usual care over 12 months from an NHS and PSS perspective as per NICE guidance.^15^ The costs from the NHS and PSS perspective comprised costs of integrated CBT and usual care, and that of healthcare and social services for mental health. Incremental costs were estimated using generalised linear regression with a gamma family and log link, adjusting for stratification (centre) and minimisation (gender and BDI-II tertiles) variables, costs of antidepressants at baseline, age and previous psychiatry referral. Incremental QALYs were estimated using mixed-effects linear regression, adjusting for all the variables above and EQ-5D-5L utility at baseline. The incremental cost-effectiveness ratio (ICER) was calculated by dividing the incremental costs by incremental QALYs when both were positive. The result was compared with the maximum acceptable ICER thresholds at £25 000–£35 000 per QALY gain recommended by NICE at the time of the analysis.^15^

Secondary analyses followed the methods of the primary analysis but were undertaken from participants’ and informal carers’ perspective and the societal perspective, respectively, to assess if the intervention had a wider impact outside of the healthcare system.^21^ The costs from participants’ and informal carers’ perspective comprised OOPs and lost income. The costs from the societal perspective comprised both the costs from the NHS and PSS perspective and from patients’ and informal carers’ perspective and the benefits received. Incremental costs from these two perspectives were estimated using generalised linear regression with gamma family and log link, adjusting for stratification and minimisation variables, along with costs of antidepressants at baseline, age, history of depression and employment status. The effectiveness measure remained QALYs and incremental QALYs were estimated as in the primary analysis. There was no recommended maximum ICER threshold for these two perspectives, the results were therefore presented without drawing conclusions.

Uncertainty around the point estimate was assessed using a non-parametric bootstrap re-sampling technique, with 5000 replicates.^22^ Multiple imputation and regression analyses, as in the primary and secondary analyses, were performed on each of the 5000 replicates. A cost-effectiveness plane (CEP) and cost-effectiveness acceptability curves^23^ were constructed using incremental estimates derived from the bootstrap iterations to illustrate the uncertainty of conclusion of cost-effectiveness.

A series of sensitivity analyses was conducted to assess the robustness of conclusions. Complete case analyses from three perspectives were undertaken following the same approach as the base case analyses but on those who had complete data on all variables involved, respectively. To examine the effect of MAR assumption, sensitivity analyses were carried out using pattern mixture modelling,^19^ assuming data were missing not at random (MNAR), that is, participants had missing values at follow-ups because they experienced ill health. Under MNAR assumption, we undertook analyses where we increased the imputed values of cost variables by 10%, 20% and 30% (incurred higher costs), reduced imputed values of EQ-5D-5L utilities by 10%, 20% and 30% (self-perceived to be in worse health), or both at the same time. A post-hoc sensitivity analysis was undertaken excluding outliers identified during the analysis process.

Excel was used for data compilation of intervention costs and data from GP records. The resulting dataset was merged with data from CRF and analysed using STATA/MP V.18.0.

## Findings

Mean age was 39.5 (SD 14.0) in the usual care group (n=226) and 39.0 (SD 15.9) in the integrated CBT group (n=225). The majority of participants were female (156 (69%) in usual care, 157 (70%) in integrated CBT). Slightly more participants in the integrated CBT group (198/225, 88%) had a history of depression prior to the study than in the usual care group (188/226, 83%). Over one-fourth of participants had a prior referral to a psychiatrist for depression (59 (26%) in usual care, 64 (28%) in integrated CBT). The proportion of participants taking antidepressants at baseline was 69% (157/226) in the usual care group and 70% (157/225) in the integrated CBT group. 57% (129/226) of the usual care group and 60% (134/225) of the integrated CBT group were in full-time or part-time employment. At 6 months, 163 participants (72.1%) in the usual care group and 171 participants (76.0%) in integrated CBT group were followed up. At 12 months, 154 participants (68.1%) in the usual care group and 173 (76.9%) in the integrated CBT group were followed up. Medical records were available for 174 usual care participants (77.3%) and 163 integrated CBT participants (72.1%).

### Costs

Nine therapists were trained and eight trainers were involved in training. Excluding research-related training, training time was estimated at 5.6 working days (7.5 hours/day) for each therapist. In most cases, training was held online (asynchronous and synchronous content) with materials provided digitally. In total, 339 weekly group supervision sessions were held. Among 225 participants allocated to integrated CBT, 137 completed therapy as planned (60.9%), 38 (16.9%) were discharged for non-attendance (therapist’s discretion under the guidance that not attending three sessions may be discharged), and 50 (22.2%) withdrew from treatment. On average, each participant attended 0.94 (SD 0.25, range 0–2) video call sessions and 5.96 (SD 3.84, range 0–13) instant messaging therapy sessions, with 1.88 (SD 1.76, range 0–9) occasions of missed/rescheduled/cancelled sessions.

Table 1 presents the breakdown of costs of integrated CBT (more detailed information see online supplemental material 2). The mean costs were estimated as £987 (SD £212) per participant.

**Table 1:** Breakdown of costs of integrated CBT (n=225)

Cost items	Costs (2022/2023) (£)
Training in use of the platform and integrated approach	
Costs of trainers	9182
Costs of therapists	23 672
Total	32 854
Per participant	146
On-going supervision	
Costs of supervisors	25 638
Costs of therapists	51 042
Total	76 680
Per participant	341
Integrated CBT delivery	
Costs of sessions per participant	412 (SD 212)
Costs of platform per participant	88
Per participant	500 (SD 212)
Costs of integrated CBT per participant	987 (SD 212)

CBT, cognitive behavioural therapy; SD, Standard deviation.

In the integrated CBT group, no participants received NHS-funded cCBT and 13 participants at 6 months and 16 at 12 months accessed NHS counselling/talking therapies. Mean costs of NHS counselling/talking therapies were £53 (SD £233, n=167) and £126 (SD £449, n=171), respectively. Mean costs of prescription of antidepressants over 12 months were £24 (SD £45, n=159).

In the usual care group, five participants accessed cCBT at 6 months (mean costs=£20, SD £114, n=158) and four at 12 months (mean costs=£19, SD £211, n=151). NHS counselling/talking therapies were accessed by 24 participants at both 6 and 12 months, with mean costs of £145 (SD £415, n=160) and £199 (SD £526, n=153), respectively. Mean costs of prescription of antidepressants over 12 months were £35 (SD £99, n=169). For details see online supplemental material 3.

Mean healthcare and social services costs were £636 (SD £1924, n=107) in the integrated CBT group and £416 (SD £633, n=111) in the usual care group. Mean OOPs were £96 (SD £364, n=127) in the integrated CBT group and £179 (SD £530, n=108) in the usual care group. Mean lost income was £1073 (SD £5164, n=103) in the integrated CBT group and £581 (SD £2132, n=91) in the usual care group. Mean benefits were £841 (SD £2348, n=99) in the integrated CBT group and £1703 (SD £6354, n=88) in the usual care group. For details, see online supplemental material 4.

### Missing data

Missing data at baseline were rare and all in the integrated CBT group (one of antidepressant costs and four of EQ-5D-5L). Missing data level for most follow-up measures was around 30%, except for participants’ days off work that reached 45%. Most missing data were due to lost-to-follow-up. The number of imputations was set to be 45. Overall missing data level at 6 and 12 months was similar. Between groups, the difference became more prominent at 12 months. Missing data due to unavailability of GP records was associated with study centre. Detailed descriptions are in online supplemental material 5.

### Primary cost-utility analysis

The primary analysis generated positive incremental costs for integrated CBT, which were mostly attributable to the treatment (table 2). Reduced usual care costs in the integrated CBT group were offset by the higher costs of healthcare and social services. The EQ-5D-5L utility was higher in the integrated CBT group than in the usual care group at all time points, resulting in higher QALYs over the trial period. The ICER lies around the middle of the range of NICE recommended £25 000–£35 000 thresholds^15^ with a low probability of integrated CBT being cost-effective over and above usual care (figure 1).

**Table 2:** Cost-utility analysis from NHS/PSS perspective: results from primary analysis and complete case analysis (sensitivity analysis)

	Primary analysis		Complete case analysis	
	Usual care (n=226)	Integrated CBT (n=225)	Usual care (n=109)	Integrated CBT (n=103)
Costs, mean (SE)				
Integrated CBT	–	£987 (£14)	–	£1051 (£17)
Usual care*	£382 (£57)	£193 (£42)	£390 (£80)	£163 (£49)
Healthcare and social services	£371 (£38)	£574 (£118)	£418 (£61)	£623 (£191)
Total NHS/PSS costs	£753 (£77)	£1754 (£127)	£808 (£115)	£1837 (£199)
EQ-5D-5L utility, mean (SE)				
Baseline	0.504 (0.017)	0.523 (0.017)	0.510 (0.024)	0.557 (0.024)
6 months	0.574 (0.021)	0.618 (0.022)	0.562 (0.026)	0.652 (0.027)
12 months	0.564 (0.022)	0.630 (0.022)	0.577 (0.028)	0.666 (0.028)
QALYs	0.554 (0.017)	0.597 (0.017)	0.553 (0.023)	0.632 (0.023)
Incremental estimates adjusted for baseline covariates†, mean (95% CI)				
Incremental costs	£1009 (£737 to £1286)		£1040 (£722 to £1462)	
Incremental QALYs	0.033 (−0.002 to 0.059)		0.046 (0.002 to 0.089)	
Incremental cost-effectiveness ratio (ICER), mean (probability of cost-effective)				
ICER	£30 576 (from £25 000 to £35 000: 26%–50%, figure 1a,b)		£22 421 (from £25 000 to £35 000: 54%–73%, figure 1c,d)	

* Usual care included NHS-funded cCBT, counselling and talking therapies, and prescription of antidepressants.

† Baseline covariates included stratification (centre) and minimisation (gender and BDI-II tertiles) variables, costs of antidepressants at baseline, age and previous psychiatry referral. Incremental QALYs were further adjusted by EQ-5D-5L utility at baseline.

CBT, cognitive behavioural therapy; EQ-5D-5L, EuroQoL 5-Domain with 5-Level; ICER, incremental cost-effectiveness ratio; NHS, National Health Service; PSS, personal social services; QALYs, quality-adjusted life years; SE, standard error.

**Figure 1:**
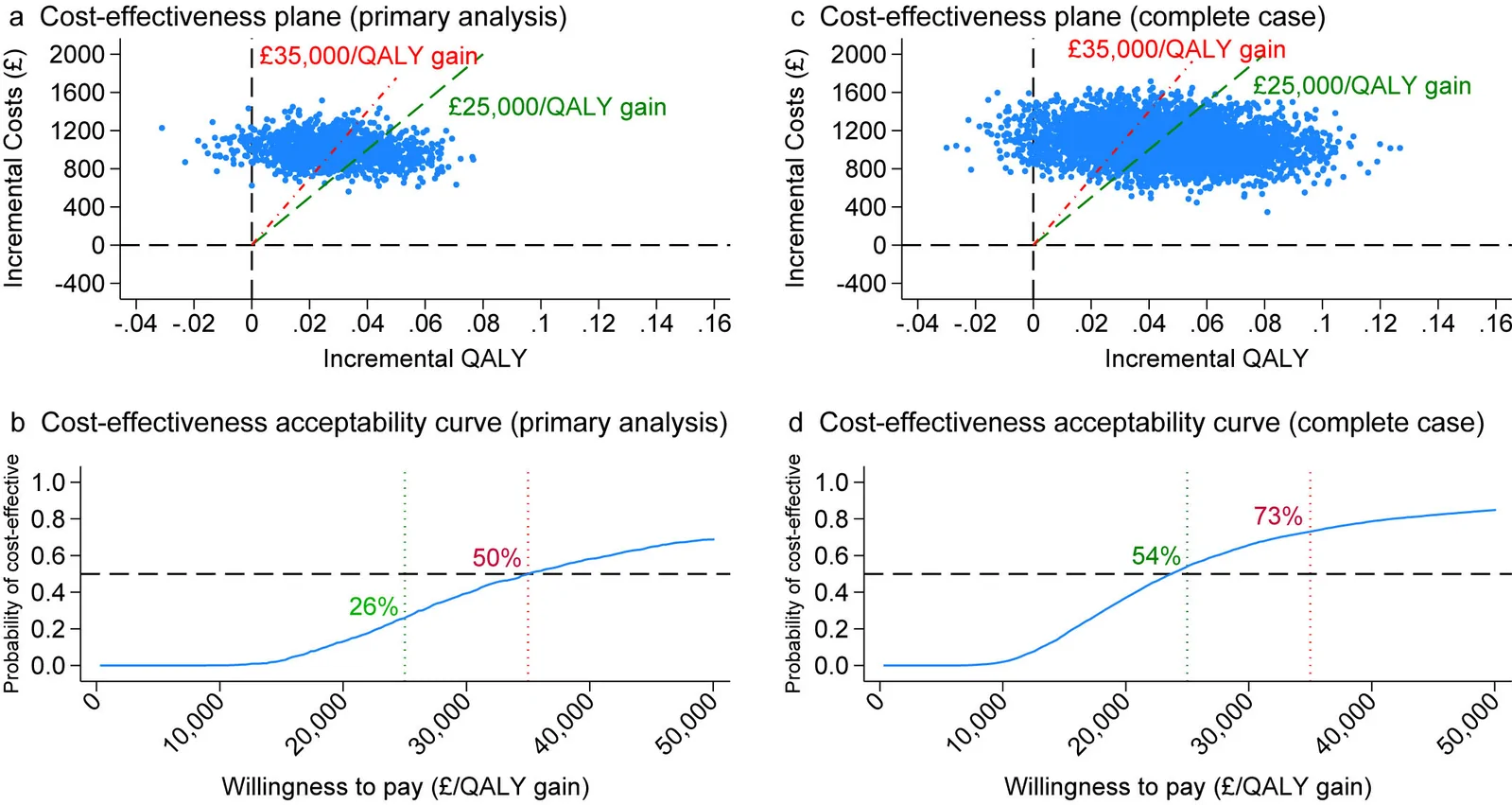
Cost-effectiveness plane and cost-effectiveness acceptability curve from National Health Service/personal social services perspective of primary analysis and complete case analysis. QALYs, quality-adjusted life years. Green line, £25 000/QALY gain. Red line, £35 000/QALY gain.

### Secondary analyses: participants and carers, and societal perspectives

From participants’ and their carers’ perspective, where the mean OOPs in the integrated CBT group were lower than in the usual care group, the lost income appeared to be in the opposite direction, especially participants’ lost income during 7–12 months (table 3). From the societal perspective, the lower benefits in the integrated CBT group were outweighed by the higher OOPs and lost income and much higher NHS/PSS costs. The CEPs from these two perspectives both show a more costly and more effective pattern (online supplemental material 6).

**Table 3:** Secondary cost-effectiveness analyses: from participants’ and their carers’ perspective and from societal perspective

	Usual care (n=226)	Integrated CBT (n=225)
From participants’ and their carers’ perspective		
OOPs and lost income, mean (SE)		
OOPs
For usual care	£110 (£22)	£62 (£20)
For healthcare and social services	£45 (£16)	£39 (£13)
Participants’ lost income		
1–6 months	£336 (£118)	£339 (£125)
7–12 months	£101 (£52)	£418 (£180)
Carers’ lost income		
1–6 months	£60 (£31)	£73 (£30)
7–12 months	£157 (£127)	£196 (£112)
Total OOPs and lost income	£808 (£190)	£1127 (£318)
Quality-adjusted life years (QALYs), mean (SE)		
QALYs	0.554 (0.017)	0.597 (0.017)
Incremental estimates adjusted for baseline covariates*, mean (95% CI)		
Incremental OOPs and lost income	£179 (−£249 to £680)	
Incremental QALYs	0.033 (−0.002 to 0.059)	
ICER	£5424 (online supplemental material 6)	
From societal perspective		
Benefits, mean (SE)		
Usual care	£390 (£63)	£150 (£34)
Other benefits	£813 (£308)	£890 (£292)
Total benefits	£1203 (£317)	£1039 (£296)
Total NHS/PSS costs	£753 (£77)	£1754 (£127)
Total OOPs and lost income	£808 (£190)	£1127 (£318)
Total societal costs	£2734 (£439)	£3890 (£475)
Quality-adjusted life years (QALYs), mean (SE)		
QALYs	0.554 (0.017)	0.597 (0.017)
Incremental estimates adjusted for baseline covariates*, mean (95% CI)		
Incremental societal costs	£1259 (£164 to £2099)	
Incremental QALYs	0.033 (−0.002 to 0.059)	
ICER	£38 152 (online supplemental material 6)	

* Baseline covariates included stratification (centre) and minimisation (gender and BDI-II tertiles) variables, and costs of antidepressants at baseline, age, history of depression and employment status. Incremental QALYs were adjusted for stratification and minimisation variables, age, previous psychiatry referral and EQ-5D-5L utility at baseline.

CBT, cognitive behavioural therapy; ICER, incremental cost-effectiveness ratio; NHS, National Health Service; OOPs, out-of-pocket payments; PSS, personal social services; QALYs, quality-adjusted life years; SE, standard error.

### Sensitivity analyses

Less than half of the participants in both groups were included in the complete case analysis from the NHS/PSS perspective. The adjusted incremental costs were similar in the primary analysis and complete case analysis, while the adjusted incremental QALYs in the complete case analysis were slightly larger than in primary analysis, resulting in a lower ICER and higher probabilities of integrated CBT being cost-effective (table 2, figure 1d).

Complete case analysis from participants’ and their carers’ perspective (n=161) yielded a negative incremental OOPs and lost income. The incremental societal costs were smaller in the complete case analysis (n=119) than in the secondary analysis. Combined with larger incremental QALYs from both perspectives, the complete case analyses generated more favourable ICERs (less costly but more effective and £10416 per QALY gain, respectively) (online supplemental material 6).

Pattern mixture modelling showed that the incremental costs became smaller with the increasing imputed values of cost variables while the incremental QALYs became larger with the reduction of imputed values of EQ-5D-5L utilities. The resulting ICERs from NHS/PSS perspective ranged from £27 027 per QALY gain to £29 588 per QALY gain (online supplemental material 7).

#### Post-hoc sensitivity analysis

During analysis, extreme values (1.5 times the next highest value) were reported by seven participants (online supplemental material 8). A post-hoc sensitivity analysis was undertaken, excluding these seven participants and re-running the multiple imputation and analysis. The results showed reduced incremental costs from all three perspectives, but only the ICER from participants’ and carers’ perspective was substantially reduced (from £5424 to £1903).

## Discussion

### Summary of main findings

From the NHS/PSS perspective, incremental costs of integrated CBT over and above usual care were mostly attributed to the costs of integrated CBT. The costliest component of integrated CBT was session delivery, followed by post-training supervision. These two components accounted for 80% of costs of integrated CBT. The ICER from the NHS/PSS perspective was just over the previous NICE threshold of £30 000 per QALY gain. By the recently updated acceptable thresholds recommended by NICE (£25 000–£35 000 per QALY gain),^15^ integrated CBT could be considered cost-effective over and above usual care although with high decision uncertainty. Sensitivity analysis using pattern mixture modelling indicated that if participants had missing values at follow-ups because they experienced ill health, the ICER would be lowered (ie, the more cost-effective integrated CBT would be).

There are no official acceptable thresholds for ICERs from a participants’ and their carers’ perspective and from a societal perspective. These would need to be determined by relevant stakeholders independently. However, from the two perspectives, QALYs might not be perceived as a straightforward effectiveness measure or a preferred one.

### Strengths and limitations of the study

This economic evaluation was part of a large multicentre RCT, which recruited to target and achieved a good follow-up rate. The sample was suitably heterogeneous and ethnically diverse, most of which were confident in using computers.^6^ We had complete data on the costs of the integrated CBT which comprised the key components for wider implementation. Over 60% of the participants completed therapy and close to 70% met the definition of compliance,^6^ which appeared lower than previous similar study.^24^ While the accuracy of usage of primary care consultations and/or the information on prescribed antidepressants was ensured by extracting them directly from participants’ GP records, the number of participants with GP records successfully retrieved was lower than those who returned self-report CRFs. This led to a higher level of missing data for primary care consultations and prescribed antidepressants. Given the high frequency of primary care use and/or antidepressant prescriptions in the study population, collecting these data by self-report over a 6-month recall period was considered less than reliable. Conversely, events associated with emergency and secondary care were much less frequent and more salient, therefore more accurate by self-report. The analyses did not adjust for costs before baseline because the data were not collected. We mitigated this by adjusting for BDI-II tertiles (severity of depression), costs of antidepressants at baseline (previous primary care access), and previous psychiatry referral or history of depression (previous healthcare resources use).

There were over 10% more missing data in information used to estimate the costs from the participants’ and their carers’, and the societal perspectives. Hence, there was greater uncertainty around estimates of cost-effectiveness from these perspectives. This greater missingness may reflect the length of the questionnaire that also collected data to enable the evaluation of clinical effectiveness on a range of outcomes. In addition, the estimation of lost income and benefits allows further uncertainty. This was by design to balance the demands of information and participant burden and the a priori decision to focus on the analysis from the NHS/PSS perspective.

While we defined usual care as NHS-funded cCBT, NHS counselling/talking therapies and antidepressants in our study, it should be noted that no restrictions were imposed to prevent participants from accessing other services available. Because the reality of usual care for depression is much more diverse and ever evolving, comparing the costs from the NHS/PSS perspective accounted for the potential costs of other usual care services beyond what was defined in the study. The costs of integrated CBT included the fixed costs of therapist training, which was estimated as 5.6 days. If the intervention were rolled out within NHS Talking Therapy services, it is likely that therapists would receive bitesize training alongside routine work rather than full-time training over a few days. However, whether shorter training would result in the same benefits in terms of clinical effectiveness remains uncertain. Furthermore, our evaluation was limited by the nature of trial setting. The fixed costs of training and supervision were not allocated to non-participating patients or carried over beyond follow-up period even if the capacity was available. Studies of CBT for patients with depression also suggested effectiveness of CBT could be maintained beyond study period.^25^ While we acknowledged possible impact on long-term costs and effectiveness, these were not included in the analysis. Taken together, these factors could make the cost-benefit of the intervention more favourable.

### Comparison with existing literature

The incremental QALYs of integrated CBT were 0.033, similar to that seen in an earlier trial of instant messaging therapy (0.034),^26^ but smaller than that in a trial of in-person CBT for patients with treatment resistant depression (0.057).^27^

Given previous evidence of cost-effectiveness of other CBT interventions for depression in similar populations,^26 27^ it was surprising that, while the integrated CBT was beneficial in terms of improvements in depressive symptoms, it was not cost-effective with high certainty under the same NICE-recommended threshold for cost-effectiveness. Hollinghurst *et al* reported an ICER of £10 083 per QALY in 2007 prices, comparing online instant messaging CBT and wait list^26^ and an ICER of £14 911 per QALY in 2010 prices when comparing in-person CBT over and above usual care.^27^ It is notable that the cost of CBT in these previous trials was lower compared with INTERACT (mean cost of online instant messaging CBT per participant: £493; mean cost of in-person CBT per participant: £910 compared with integrated CBT: £987). While some of that was attributed to our inclusion of training and routine supervision costs and the cost of maintaining the therapy platform in estimating intervention costs, inflation from 2007 to 2022/2023 (approximately 50% increase in the health sector^10^) is also a contributory factor.

Notably, in 2022/2023, NHS Talking therapies services provided on average 8.1 sessions of treatment per patient.^28^ Multiplying by the unit cost of £159 per care contact,^29^ the estimated costs per patient amount to £1288, which is higher than the cost of integrated CBT (£987) in this RCT.

## Clinical implications

While the integrated CBT was found clinically effective at 6 months and maintained improvements at 12 months in our trial,^6^ and the intervention costs were favourable compared with the average cost of a course of CBT delivered in NHS talking therapy services, the cost-effectiveness over and above usual care remained inconclusive over a 12-month time horizon. Evidence is needed to substantiate and quantify any long-term gain for this novel integrated approach. While participants who were more comfortable communicating with little in-person contact found integrated CBT acceptable and helpful in allowing more time to organise their thoughts, many participants struggled to express themselves through text-based communication and sometimes found it anxiety provoking.^7^ More broadly, digital exclusion remains a challenge across the UK, where barriers such as device poverty, unaffordable internet access, and low digital literacy or confidence persist, especially among older populations or deprived households.^30^ Carefully designed digital exclusion assessment should be considered locally before adopting digital interventions to avoid widening health inequalities.

## Supplementary material

10.1136/bmjment-2026-302884 online supplemental file 1

## Data Availability

Data are available upon reasonable request.
