# Supplementary material for: Cost-utility analysis of integrated therapist and online CBT over and above usual care for patients with depression in primary care: an economic evaluation of the INTERACT randomised controlled trial
Source: BMJ Ment Health. 2026 Oct 22;29(1):e302884. doi: 10.1136/bmjment-2026-302884 (PMC13691772; doi:10.1136/bmjment-2026-302884)
Supplement: Supplementary file 1 [file bmjment-29-1-s001.pdf]

Tallon et al. Trials (2023) 24:421
https://doi.org/10.1186/s13063-023-07396-9

Trials

STUDY PROTOCOL

Open Access

Integrated therapist and online CBT for depression in primary care (INTERACT): study protocol for a multi-centre randomised controlled trial

Debbie Tallon[1*], Laura Thomas[1], Sally Brabyn[2], Brian Chi Fung Ching[3], Jane Sungmin Hahn[3], Berry Jude[3], Mekeda X Logan[3], Alex Burrage[1], Fiona Fox[1], Simon Gilbody[4], Paul Lanham[5], Glyn Lewis[3], Jinshuo Li[6], Stephanie J. MacNeill[7], Irwin Nazareth[8], Steve Parrott[6], Tim J. Peters[9], Roz Shafran[10], Katrina Turner[11], Chris Williams[12,13], David Kessler[1†] and Nicola Wiles[1†]

Abstract

Background Cognitive behavioural therapy (CBT) is an effective treatment for depression. Self-directed online CBT interventions have made CBT more accessible at a lower cost. However, adherence is often poor and, in the absence of therapist support, effects are modest and short-term. Delivering CBT online using instant messaging is clinically and cost-effective; however, most existing platforms are limited to instant messaging sessions, without the support of between-session "homework" activities. The INTERACT intervention integrates online CBT materials and 'high-intensity' therapist-led CBT, delivered remotely in real-time. The INTERACT trial will evaluate this novel integration in terms of clinical and cost-effectiveness, and acceptability to therapists and clients.

Methods Pragmatic, two parallel-group multi-centre individually randomised controlled trial, with 434 patients recruited from primary care practices in Bristol, London and York. Participants with depression will be identified via General Practitioner record searches and direct referrals. Inclusion criteria: aged ≥ 18 years; score ≥ 14 on Beck Depression Inventory (BDI-II); meeting International Classification of Diseases (ICD-10) criteria for depression. Exclusion criteria: alcohol or substance dependency in the past year; bipolar disorder; schizophrenia; psychosis; dementia; currently under psychiatric care for depression (including those referred but not yet seen); cannot complete questionnaires unaided or requires an interpreter; currently receiving CBT/other psychotherapy; received high-intensity CBT in the past four years; participating in another intervention trial; unwilling/unable to receive CBT via computer/laptop/smartphone. Eligible participants will be randomised to integrated CBT or usual care. Integrated CBT utilises the standard Beckian intervention for depression and comprises nine live therapist-led sessions, with (up to) a further three if clinically appropriate. The first session is 60–90 min via videocall, with subsequent 50-min sessions delivered online, using instant messaging. Participants allocated integrated CBT can access integrated online CBT resources (worksheets/information sheets/videos) within and between sessions.

†David Kessler and Nicola Wiles are joint senior authors.

*Correspondence:
Debbie Tallon
d.tallon@bristol.ac.uk
Full list of author information is available at the end of the article

© The Author(s) 2023. **Open Access** This article is licensed under a Creative Commons Attribution 4.0 International License, which permits use, sharing, adaptation, distribution and reproduction in any medium or format, as long as you give appropriate credit to the original author(s) and the source, provide a link to the Creative Commons licence, and indicate if changes were made. The images or other third party material in this article are included in the article's Creative Commons licence, unless indicated otherwise in a credit line to the material. If material is not included in the article's Creative Commons licence and your intended use is not permitted by statutory regulation or exceeds the permitted use, you will need to obtain permission directly from the copyright holder. To view a copy of this licence, visit http://creativecommons.org/licenses/by/4.0/. The Creative Commons Public Domain Dedication waiver (http://creativecommons.org/publicdomain/zero/1.0/) applies to the data made available in this article, unless otherwise stated in a credit line to the data.

Outcome assessments at 3-, 6-, 9- and 12-month post-randomisation. The primary outcome is the Beck Depression Inventory (BDI-II) score at 6 months (as a continuous variable). A nested qualitative study and health economic evaluation will be conducted.

Discussion If clinically and cost-effective, this model of integrated CBT could be introduced into existing psychological services, increasing access to, and equity of, CBT provision.

Trial registration ISRCTN, ISRCTN13112900. Registered on 11/11/2020. Currently recruiting participants. Trial registration data are presented in Table 1.

Keywords Depression, Randomised controlled trial, Cognitive behavioural therapy, Internet-based treatment, Blended treatment, Primary care, Integrated cognitive behavioural therapy, Acceptability, Qualitative research

Background

Cognitive behavioural therapy (CBT) is an effective treatment for depression [1, 2]. Substantial investment in NHS Talking Therapies [3] (formerly known as Improving Access to Psychological Therapies (IAPT) services [4]) has increased provision of CBT for depression; however, there is still considerable geographical variation in the provision of therapy [5] (Table 1).

Computerised CBT interventions (cCBT) were designed to make CBT more accessible and widely available at a lower cost. However, adherence to cCBT is often poor and, in the absence of therapist support, effects are modest and short-term [6]. Moreover, cCBT is often inflexible and does not allow identification of conditional beliefs or detailed formulations [7], yet the latter are crucial elements of CBT.

Therapist-delivered online CBT for depression using instant messaging is clinically and cost-effective [8, 9]. However, most existing platforms are limited to instant messaging sessions only, without the support of "homework" activities between sessions. Moreover, they also depend on the access to online sessions via a desktop computer, which can be a barrier to those who do not have access to a computer or share one with relatives, partners or friends. Making the platform also available on mobile devices could address these barriers, especially given that the use of smartphones to access the internet has greatly increased over the last 15 years: as of 2021, the vast majority (88%) of UK adults owned a smartphone [10]. Therefore, interventions that incorporate online resources accessed via mobile devices may reach more effectively into people's lives, allowing more immediate recording of thoughts and experiences. Mobile devices may also enable discreet and convenient completion of worksheets (such as thought diaries) thereby addressing concerns about the risk of "getting caught" completing homework tasks in public [11]. Use of such devices may therefore increase engagement with CBT "homework" which is an important mediator of outcome [12, 13].

The INTERACT study is an 8-year programme of research funded by the National Institute for Health and Care Research (NIHR) Programme Grants for Applied Research that aims to integrate online CBT materials and high-intensity therapist-led individual CBT, delivered remotely in real-time by an accredited therapist, to people with depression. If effective, this form of integrated CBT could reduce costs and increase availability of CBT for people with depression, including those for whom access is difficult (e.g. working full-time/living in remote areas/with caring responsibilities and hard-to-reach groups).

The INTERACT research programme brings together a large multi-disciplinary team, including mental health academics and practitioners, human–computer interaction researchers, software engineers and end users. It consists of three stages. The first stage focused on: identifying clinical and cost-effective components of CBT to inform the design of the intervention [14, 15] development of an online platform for delivering integrated high-intensity CBT for depression and gathering additional design ideas [16, 17] and feedback from key stakeholders such as CBT therapists [18, 19] and patients with experience of CBT [20]. The second stage involved a pilot evaluation of the therapy platform — delivering CBT to 17 primary care patients with depression, using the platform, and gathering detailed qualitative and technical feedback from patients and the therapists delivering the intervention [21]. This led to further refinements of the online platform, the integrated CBT resources, and training materials for therapists who will deliver the integrated intervention. This novel integration, which blends high-intensity therapist-led CBT with innovative use of technology, now needs to be evaluated in terms of clinical and cost-effectiveness, and acceptability, to inform policy and practice. Therefore, this third and final phase of the INTERACT programme is a multi-centre randomised controlled trial (RCT) to fully evaluate the clinical and cost effectiveness of this integrated approach to CBT for depression. This protocol relates to the RCT evaluation.

Prior to the start of this programme, no registered trials [22] integrated online materials with therapist-led CBT for depression. An EU project (E-COMPARED)

Table 1 Trial registration data set

Data category	Information
Primary registry and trial identifying number	ISRCTN13112900
Date of registration in primary registry	11/11/2020
Secondary identifying numbers	National Institute for Health and Care Research (NIHR) Portfolio ID 41172
Source(s) of monetary or material support	NIHR Programme Grants for Applied Research
Sponsor	Mr Adam Taylor<br>University of Bristol, Research and Enterprise Development (RED), One Cathedral Square, Bristol, BS1 5DD<br>Email: research-governance@bristol.ac.uk
Contact for public queries	Email: bris-interact@bristol.ac.uk
Contact for scientific queries	Email: bris-interact@bristol.ac.uk
Public title	Integrated CBT for depression trial (INTERACT RCT)
Scientific title	Multi-centre randomised controlled trial of integrated therapist and online CBT for depression in primary care (INTERACT)
Countries of recruitment	England
Health condition studied	Depression
Trial participants	Primary care patients with depression
Intervention(s)	Integrated therapist and online cognitive behavioural therapy for depression
	Usual GP care, which may include referral to Improving Access to Psychological Therapy (IAPT) services or antidepressant medication as appropriate
Key inclusion and exclusion criteria	Ages eligible for study: ≥ 18 years<br>Sexes eligible for study: both<br>Accepts healthy volunteers: no
	Inclusion criteria:<br>Aged ≥ 18 years<br>Scores ≥ 14 on Beck Depression Inventory (BDI-II)<br>Meets ICD-10 criteria for a primary diagnosis of depression
	Exclusion criteria:<br>Alcohol or substance dependency in the past year<br>Bipolar disorder<br>Schizophrenia/psychosis<br>Dementia<br>Currently under psychiatric care (including those referred but not yet seen) for depression<br>Cannot complete questionnaires unaided or would require an interpreter<br>Are currently receiving CBT or other psychotherapy<br>Have received high-intensity CBT in the past 4 years<br>Are taking part in another intervention trial<br>Not willing or able to receive CBT via computer/laptop/smartphone
Study type	Pragmatic, two parallel-group multicentre randomised controlled trial (RCT) with allocation at the level of the individual
	Allocation: randomised
	Primary purpose: treatment
	Phase III trial, with nested qualitative study and health economic evaluation
Date of first enrolment	7/1/2021
Target sample size	434
Recruitment status	Recruiting
Primary outcome(s)	Score on the Beck Depression Inventory (BDI-II) at 6 months (measured as a continuous variable)

Table 1 (continued)

Data category	Information
Key secondary outcomes	Secondary outcomes include:<br>Response defined as at least 50% reduction in depressive symptoms on the BDI-II at 6 months compared with baseline<br>Remission of depressive symptoms (BDI-II < 10) at 6 months<br>Percentage reduction in depressive symptoms on the BDI-II at 6 months<br>Depressive symptoms on the PHQ-9 at 6 months<br>Quality of life using the WSAS and EQ-5D-5L at 6 months<br>Anxiety symptoms (GAD-7) at 6 months<br>All of the above primary and secondary outcomes measured at 12 months post-randomisation<br>Use of primary care consultations and prescribed medication (from GP records over 12 months)<br>Use of other primary and community care services; secondary care related to mental health, private treatments, use of social services, burden on informal caregivers, personal costs related to mental health and benefits received (questionnaire at 6 and 12 months post-randomisation)

of 'blended care' (face-to-face CBT and self-help) for depression [23] has some similarities but, in our model, therapist contact takes place in real-time via instant messaging rather than in-person, and the online materials (e.g. worksheets to help the patient gather information and try out what they are learning in practice) are an integral part of therapy. A new trial has been registered recently (ISRCTN11129335) that will investigate blended CBT for depression (in-person CBT supported by a smartphone app (Elona therapy)). However, the major difference compared with this study and E-COMPARED is that we will compare our intervention with usual GP care, which may include including referral to NHS Talking Therapies (formerly known as IAPT services) or antidepressant medication as appropriate. In contrast, E-COMPARED is a non-inferiority comparison between 'blended care' and in-person CBT, and the comparison group for the Elona therapy trial is also in-person CBT. Given the continued difficulty in accessing in-person individual CBT in the UK with less than one-third of those with depression being seen in IAPT services by 2023/24 [24], neither trial answers a pragmatic question for UK primary care. In addition, data from other countries may not generalise more widely and a UK trial will provide cost-effectiveness data, which are required to inform the provision of NHS services. If clinically and cost-effective, this model of integrated CBT could easily be introduced into existing psychological services, increasing access to, and equity of, CBT provision.

Objectives

To establish (1) the clinical effectiveness and (2) cost-effectiveness of an integrated approach to delivering CBT in reducing depressive symptoms and improving quality of life over 12 months (compared with usual care) for primary care patients with depression.

In addition, a nested qualitative study will allow us to (1) explore patients', therapists' and supervisors' views and experiences of using an integrated approach to delivering CBT; (2) understand patients' reasons for completing or not completing treatment; and (3) assess patients', therapists' and supervisors' views on how this novel approach affects the therapist-patient relationship.

Methods

Design

Pragmatic, two parallel-group, multi-centre, superiority randomised controlled trial, with 1:1 allocation at the level of the individual. The trial design is summarised in Fig. 1. The trial protocol was written according to the Standard Protocol Items: Recommendations for Interventional Trials Statement (SPIRIT) [25]. A SPIRIT table is provided in Fig. 2 and a SPIRIT checklist is included in Additional file 1.

Trial setting

The study will be based in primary care in three trial centres: the University of Bristol (coordinating centre), University College London, and the Universities of Hull/York. Patients will be recruited from primary care (General Practices) set in the surrounding areas of Bristol, London and Hull/York. General Practices will act as Participant Identification Centres (PICs).

Participant eligibility criteria

Inclusion criteria

Aged ≥ 18 years; score ≥ 14 on Beck Depression Inventory (BDI-II)**;** meet ICD-10 criteria for a primary diagnosis of depression (assessed using the Clinical Interview Schedule – Revised version (CIS-R) [26, 27]).

Exclusion criteria

Alcohol or substance dependency in the past year; bipolar disorder; schizophrenia; psychosis; dementia; currently under psychiatric care (including those referred but not yet seen) for depression; cannot

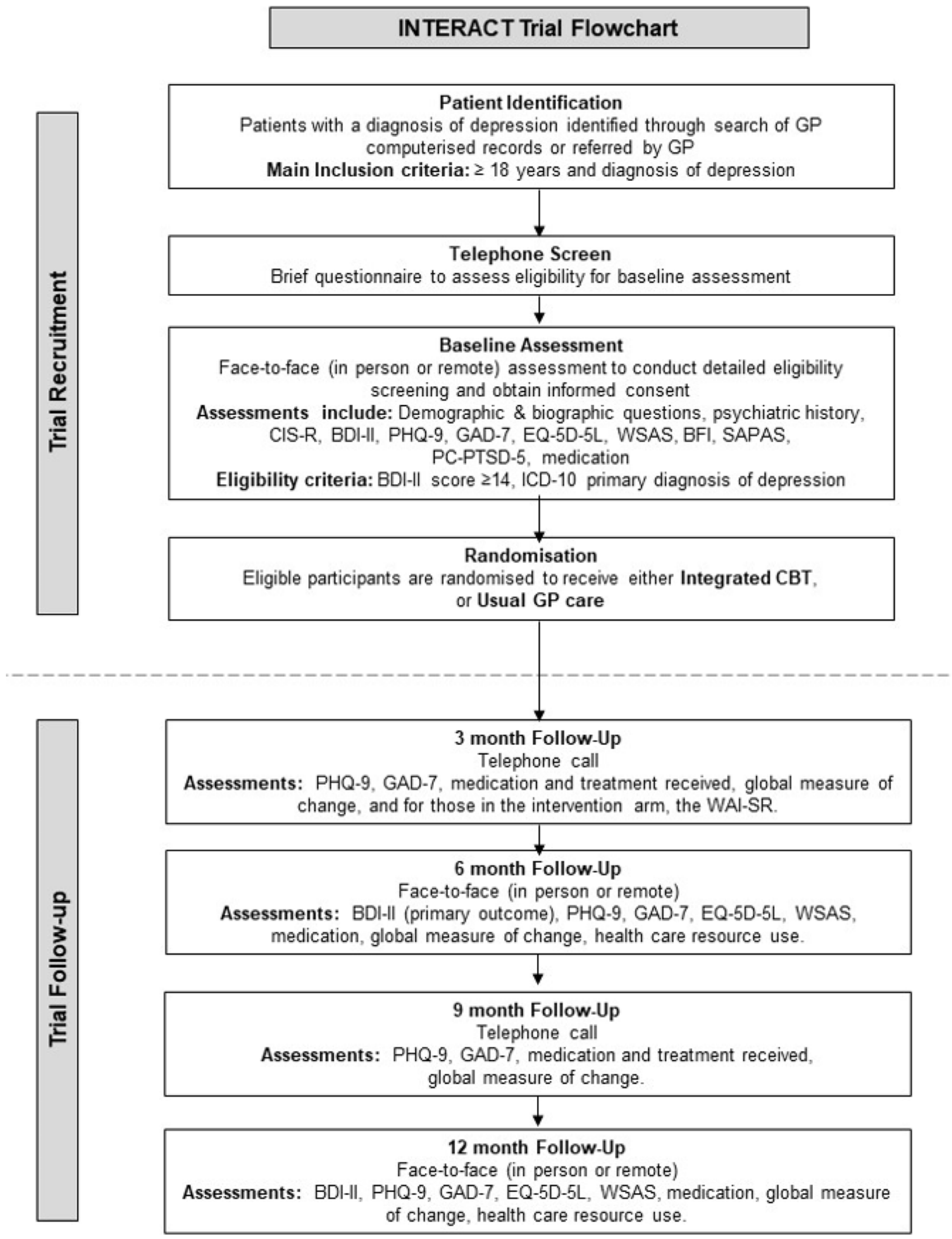

Fig. 1 INTERACT trial flowchart

complete questionnaires unaided or would require an interpreter; are currently receiving CBT or other psychotherapy; have received high-intensity CBT in past 4 years; are taking part in another intervention trial; not willing or able to receive CBT via computer/laptop/smartphone.

These broad inclusion criteria will result in a heterogeneous population (those with a new episode of depression who prefer psychological treatment, those who have not responded to initial treatments, and those with severe/chronic depression). These are all groups for whom psychological interventions for depression are effective and are recommended by National Institute for Health and Care Excellence (NICE) [28].

Trial procedures

Recruitment

The trial aims to recruit participants with depression from primary care. We will use two methods of recruitment — namely, record search and in-consultation recruitment. Practices will be given the option of displaying an INTERACT poster on their premises. Recruitment will take place in general practices linked to the three trial centres and will be supported

	STUDY PERIOD						
	Telephone Screen	Baseline	Post-allocation (months)				Close-out
**TIMEPOINT**	Pre-baseline	0	3	6	9	12	After 12-months
**ENROLMENT:**							
**Telephone eligibility screen**	X						
**Informed consent**		X					
**Baseline eligibility screen**		X					
**Allocation**		X					
**INTERVENTIONS:**							
Integrated CBT*							
Usual Care							
**ASSESSMENTS:**							
**Telephone screen:** Age, gender, current psychological therapy, ability to complete questionnaires unaided, participation in other research, receipt of CBT in the past 4 years,	X						
able to receive online CBT, bipolar disorder/schizophrenia/psychosis/ dementia, alcohol/drug dependence in past year							
Biographic and demographic data (e.g. employment status, qualifications, ethnicity, housing status, marital status, history of depression), IT Literacy		X					
Life events, Social Support, Alcohol use (AUDIT-PC), Financial status, BFI Neuroticism, SAPAS, PC-PTSD-5		X*					
CIS-R		X					
BDI-II		X		X		X	
EQ-5D-5L, WSAS		X*		X		X	
PHQ-9, GAD-7		X*	X	X	X	X	
Antidepressant medication and adherence		X	X	X	X	X	
Global measure of change			X	X	X	X	
Your INTERACT Therapy**			X	X	X	X	
Devices used to access platform**			X	X	X	X	
WAI-SR**			X				
WAI-SRT** (completed by therapist)			X				
Therapy receipt (outside of trial)			X	X	X	X	
Health care resource use				X		X	
Exit questionnaire						X	
Primary care consultations and prescribed medication (from primary care notes)							X

Fig. 2 Schedule of enrolment, interventions and assessments. *Eligible participants only. **Intervention arm participants only. ***9 sessions of integrated CBT with (up to) a further 3 if deemed clinically necessary; access to the platform CBT resources for 12-months post-randomisation

by well-established local NIHR Clinical Research Networks (CRNs). The planned recruitment period is 21 months, with a target of approximately 8 participants randomised per centre per month.

Participant identification

Method 1: record search GP practices will conduct a search of their computerised records for potentially eligible patients (defined as those who are aged 18 years or over, who have been diagnosed with depression). Practices will be asked to exclude those who would be unsuitable due to the exclusion criteria. The record search will be conducted using primary care diagnostic codes. Practices will be given the opportunity to manually screen the resulting list.

Potentially eligible patients will then be mailed an invitation to participate by the GP practice, asking for their permission to be contacted by the research team. Patients who have not responded after two weeks will be sent one reminder letter by the practice.

Patients will be able to respond anonymously if they wish to decline participation, and will be able to provide a reason for declining. Decliners will be asked to indicate whether they are willing to be interviewed briefly over the telephone about their reasons for declining, and if so to add their contact details to the form. We will also ask practices to provide anonymised data for all patients identified by the record search (age, gender, reason for exclusion by practice). These data will be used to report the generalisability of results.

Method 2: in consultation General Practices (Participant Identification Centres) can identify patients in consultation that they think might be suitable for the trial. They will introduce the trial and ask the patient for their permission to be contacted by the research team (verbal consent can be taken during telephone and videocall consultations).

Potential participants who agree to contact via either recruitment method (letter/in consultation) will be telephoned by the local research team and asked a few brief screening questions to confirm their suitability for a baseline appointment. Those meeting the telephone screening criteria would then be invited to an appointment with a researcher to explain the trial, perform the baseline assessment, establish eligibility and obtain written informed consent.

Telephone screening

Primary care patients who have been referred, or expressed interest by returning their postal invitation reply form, will be telephoned by a researcher from the local site. The researcher will briefly explain the study, check that a patient information sheet has been received, and answer any questions the participant may have.

With verbal consent, the researcher will then proceed with the telephone screening questionnaire. This will include questions about demographic information and the main exclusion criteria. The screening call is likely to take 10–15 min. If telephone screening is not practical for the participant (e.g. if they are hard of hearing) we will make adaptations where possible (e.g. offer screening via post/email). A list of study questionnaires is presented in Fig. 2 (SPIRIT table).

If a participant meets the screening criteria, they will be offered a detailed eligibility screening appointment (baseline assessment) with the researcher.

Baseline assessment

This appointment will last 1–1.5 h and will take place in-person at the patient's home, GP surgery, the recruiting University's premises, or other mutually convenient location, where it is safe to do so. Alternatively, the appointment will take place remotely, with the patient completing online questionnaires on their own smartphone, tablet or computer, and the researcher providing support via telephone or videocall. The local researcher will explain the study in more detail, answer questions the participant may have, and obtain written informed consent. The researcher will check whether the participant's circumstances have changed since they completed the screening questionnaire (for example if they have subsequently started to receive therapy or been referred to secondary care) to check they are still eligible for a baseline assessment.

Potentially eligible participants will then be asked to complete a number of questionnaires. These will include the Beck Depression Inventory (BDI-II) [29] (a brief measure of depressive symptoms), and the Clinical Interview Schedule – Revised version (CIS-R) [26, 27], an in-depth self-assessment of psychiatric symptoms that establishes whether an ICD-10 diagnosis of depression is met. Sociodemographic questions will include age, gender, employment status, qualifications, ethnicity, housing and marital status. Participants will also be asked for relevant medical history including co-morbidity [30] and history of depression and treatment including antidepressant medication and adherence. They will be asked if they are willing for their summary data to be passed to their GP.

Participants who score 14 or more on the BDI-II and have an ICD-10 primary diagnosis of depression on the CIS-R will be told they are eligible to enter the trial. Eligible participants will be asked to complete further questionnaires including the Patient Health Questionnaire (PHQ-9) [31] and General Anxiety Disorder questionnaire (GAD-7) [32], which are brief measures of depression and anxiety used in psychological services; the EQ-5D-5L [33] and the Work and Social Adjustment Scale (WSAS) [34], a simple measure of impairment in functioning. They will also be asked to complete: the Big Five Personality Inventory (BFI) neuroticism subscale [35]; a personality scale (SAPAS) [36]; and a measure of trauma (PC-PTSD-5) [37]. Eligible participants will be asked further questions about their history of depression and whether they have ever been referred to a psychiatrist. Additional information will be collected on life events, financial stress, social support and alcohol use [38].

Arrangements for starting therapy will be discussed with those allocated to receive the intervention and these individuals will also be shown how to log onto the therapy platform. The researcher will provide a brief client handout which summarises policies regarding confidentiality, cancelling or rescheduling sessions, contacting the therapist between sessions, safety, contact for technical queries, and downloading materials from the platform.

Re-screening

Patients who do not meet the eligibility criteria at telephone screen or baseline may be eligible for rescreening. For example, those who are currently receiving a course of therapy could be rescreened once this course has ended. Patients who do not meet the BDI-II or ICD-10 criteria at baseline could be offered a rescreen as long as one month had elapsed since their original baseline appointment and the study was still recruiting.

Informed consent for trial participation

Prior to commencing the baseline assessment, the researcher will obtain written informed consent from the patient relating to their participation in the trial, including consent to be approached regarding a qualitative interview. Informed consent will be obtained either via an online consent 'e-consent' method (if the baseline assessment is being conducted remotely by telephone or videocall) or via paper-based informed consent if the researcher is meeting with the patient face-to-face. In either case, patients will be able to ask the researcher questions prior to consenting. Patients attending a face-to-face appointment will be given a copy of their written consent form to keep and patients completing the assessment remotely will receive an electronic copy of their e-consent form via email. A copy will also be sent to the patient's GP if they are eligible to take part in the study.

Participants will be reminded that they are free to withdraw from the trial at any time without giving reasons and without prejudicing their further treatment. We will seek consent to use data collected up to the point of withdrawal, and this will be explained in the information sheet. In addition, in line with open-access data requirements, information may also be used to support other research in the future and may be shared anonymously with other researchers. This will be explained in the information sheet.

Additional consent provisions for collection and use of participant data in ancillary studies

As part of the baseline consent procedure, patients who give informed consent for trial participation will confirm they understand their data may be used to support other research in the future and may be shared anonymously with other researchers. Trial participants will also indicate whether they would be willing to be contacted about future related research.

Optional supplementary study (Bristol participants only)

Eligible participants who are recruited by the University of Bristol site will be asked if they would be interested in hearing more about an *optional* supplementary scanning study, 'BRAINTERACT' (IRAS Study ID: 260380), which would involve attending one functional magnetic resonance imaging (fMRI) scan at the Cardiff University Brain Research Imaging Centre (CUBRIC) in Cardiff. If the participant is potentially interested in this fMRI study, the researcher will provide a full BRAINTERACT participant information sheet and will seek permission (written or verbal) to pass the participant's contact details to the BRAINTERACT researcher so that they can provide more details. This supplementary study is detailed in a separate protocol and is funded by the NIHR Bristol Biomedical Research Centre.

Randomisation

Randomisation will be stratified by centre and minimised on gender (male/female), current antidepressant use (yes/no), and depression severity using BDI-II tertiles derived from the CoBalT study [2, 39] baseline scores (BDI-II $\leq$ 25; 26–35; $\geq$ 36). If participants indicate a non-binary gender at baseline, we will use an unpredictable computer-generated code to randomly select a binary gender code, prior to randomising them to a treatment group in the usual way. (Details of self-reported gender will be given in full as part of the reporting process.) We will use minimisation with a probability weighting of 0.8 to reduce predictability [40]. Stratifying by centre will

ensure a balance in terms of local differences and also proportionate workload for therapists. The minimisation variables are important prognostic indicators on which we wish to ensure a balance between the two groups.

Method of implementing the randomisation/allocation sequence

The randomisation scheme will be developed independently by the Bristol Trials Centre (BTC) on behalf of the study. BTC will use a computer-generated code to produce a 1:1 randomisation sequence. This will be unpredictable and concealed from the study team by use of an automated, remote randomisation service, which is accessible by telephone or online.

Once eligibility has been confirmed at the baseline assessment, the researcher will enter the patient's details onto BTC's secure, 24-h randomisation system, either by telephone or online.

Once the details required for randomisation have been confirmed, the remote system will immediately reveal the allocation (integrated CBT intervention or usual care) to the research team. The researcher will inform the patient of the allocation during the baseline appointment where possible, or by telephone/email shortly afterwards.

Confirmation of the patient's treatment allocation will be automatically and immediately emailed to the coordinating centre. The allocation will be recorded on the trial database. The local research team will send written confirmation of the allocation to the patient's GP.

Blinding

It will not be possible to blind participants to their treatment allocation because of the nature of the intervention. To eliminate the potential for observer bias, we will use self-reported outcome measures (e.g. BDI-II) to assess outcomes. These outcomes have been widely used in previous depression trials [2, 8, 41]. We are not using clinician-based measures of outcomes, such as the Hamilton Rating Scale for Depression [42], as maintaining blinding would be difficult and thus the outcome assessment would be at a higher risk of bias.

Follow-up assessments

Trial follow-up assessments will be conducted at 3 months, 6 months, 9 months and 12 months after randomisation. Participants will be followed up face-to-face (in person or via videocall) at 6 and 12 months, with the other follow-ups taking place over the telephone, online, or via videocall. A summary of the assessments is presented in Fig. 2.

Three- and 9-month post-randomisation

Where possible, the researcher will contact the participant by telephone (10–15 min) to conduct the follow-up questionnaire. Alternatively, the follow-up may be conducted using online questionnaires, by videocall, or by post. The patient will complete PHQ-9 and GAD-7 questions and brief questions about antidepressant medication. They will be asked to indicate how their symptoms have changed in the last 3 months and to report any therapy received outside of the trial. Intervention participants will be asked some questions about their receipt of integrated therapy. At 3 months, intervention participants will also complete the WAI-SR [43] and their therapist will complete the therapist version of the Working Alliance Inventory (WAI-SRT) [43].

Six- and 12-month post-randomisation

Where possible, the researcher will meet the participant face-to-face (in person or via videocall) to complete this 30-min assessment (otherwise it will be conducted by telephone, online or post). The participant will complete questionnaires including the BDI-II (primary outcome), PHQ-9, GAD-7, EQ-5D-5L, and WSAS. They will also be asked about their use of antidepressant medication, and use of health care resources in the past 6 months and to indicate how their symptoms have changed in the last 3 months.

After the 6-month follow-up, interviews with those taking part in the nested qualitative study will take place. At the 12-month follow-up, participants will be asked to complete an 'exit questionnaire' about their reasons for taking part in the study, and their experience of taking part. They will be asked if they would be willing to be interviewed about their reasons for taking part, and their experiences. 'Exit interviews' will take place after the 12-month follow-up.

Retention strategies

Participants are offered £10 vouchers as a 'thank you' for the 3-, 6- and 12-month follow-up, and a for a qualitative interview. Adaptations to mode of communication are possible (e.g. videocall, telephone, post, online survey). Remote appointments (e.g. via videocall) were introduced due to the Covid pandemic and continued to be offered after this. If full questionnaire data collection is not feasible, the researcher will attempt to collect the BDI-II, current medication and adherence, and receipt of other psychological treatments as a minimum.

Withdrawal of trial participants

Patients who withdraw from the trial intervention will be asked if they are still willing to provide follow-up data

and/or take part in a qualitative interview if sampled. Participants have the right to withdraw from the trial at any time for any reason, without their medical care being affected. If a patient in either arm of the study withdraws from the study completely, no further data will be collected — this includes the collection of health resource use data from the patient's medical notes at the end of the trial. If a patient withdraws, the reason for and type of withdrawal will be documented in the case report form (CRF). Where possible, data collected prior to withdrawal will continue to be used in the trial.

Participants who commence concurrent psychological therapy after randomisation to the intervention arm will *not* be withdrawn from the intervention or study by the trial team, though they would be advised against commencing concurrent therapy. Patients in the intervention arm will *not* be withdrawn from the intervention or study due to lack of efficacy.

Ancillary and post-trial care

We will inform the patient's GP when they withdraw or complete their participation in the study. Those in the intervention arm will receive usual care from their GP following the completion of therapy. Appropriate legal liability insurance is in place for the trial.

Trial intervention and comparator

Participants will be randomised to either receive the intervention — integrated CBT, or to continue with usual care from their GP.

Trial intervention — integrated cognitive behavioural therapy for depression

The intervention is provided individually and comprises nine therapist-led sessions, with (up to) a further three sessions if deemed clinically appropriate by the therapist. The first session will last 60–90 min and take place face-face via videocall (independent of the INTERACT platform), with subsequent 50-min sessions taking place online using the INTERACT therapy platform, via instant messaging/typing. Patients in the intervention arm will also continue to be cared for as usual by their GP.

The INTERACT protocol utilises the standard Beckian intervention for depression [44-46]. In considering the detailed content of Beck's intervention for depression, we refer to the UCL competence framework for CBT [47] and the problem-specific competences [48].

To ensure that the results of the trial apply to standard clinical settings, we will recruit CBT practitioners comparable to those working as high-intensity therapists within IAPT services. Hence, therapists will be mental health professionals with appropriate post-qualification CBT training (British Association for Behavioural and Cognitive Psychotherapies (BABCP) accreditation) or equivalent experience. All therapists will be employed by the study or seconded to the study from local NHS Trusts/Psychological Services, for the duration of the study. Therapists will start 1 month before the start of delivery of therapy in order for them to receive training in the use of the platform and study procedures, and to enable them to familiarise themselves with the range of resources available. During the study, therapists will receive weekly supervision from an experienced therapist. This arrangement mimics local NHS practice and meets the standards for clinical supervision set out by the BABCP. Therapists will be provided with detailed training manuals and SOPs, e.g. for risk assessment and management.

Prior to the start of therapy, the researcher will give the participant a brief demonstration of the platform and its key features. Participants will also be sent a brief handout which summarises policies regarding client confidentiality, cancelling or rescheduling sessions, contacting the therapist between sessions, safety, contact for technical queries, and downloading materials from the platform as well as a user guide to the platform, which outlines key features and tips for use. They will receive login details to the platform before the first session to enable them to create an account and familiarise themselves with the platform prior to this first session. They will also be asked to provide some brief details about the problems that bring them to therapy, previous treatment, depressive symptoms (using the PHQ-9) and to plan for their first session within the platform. The therapist will be able to answer any queries regarding the platform during the first session. Participants will be able to view the various psychoeducational resources (e.g. information sheets and short animations) within the platform once they log in but other worksheets/resources will be released as appropriate (tailored to the individual) by the therapist during treatment.

The first therapy session will take place face-to-face via videocall (independent of the INTERACT platform) and may take up to 90 min. This longer duration will enable the completion of history taking, introduction of the CBT model and other relevant psychoeducation. In addition, therapists will help the patient become familiar with the INTERACT platform.

Subsequent therapy sessions will take place online using the INTERACT platform and will usually last 50 min. The platform enables the patient and therapist to communicate online in real-time and to use a collaborative workspace to view, edit and discuss CBT resources within the platform. Online communication will take place using instant messaging and may also involve the

telephone. As per standard in-person CBT, therapists and patients will agree tasks ("homework") for the patient to try between therapy sessions. These tasks will utilise materials embedded within the INTERACT platform, such as interactive CBT worksheets which can be typed into, and other psychoeducational materials. Examples of completed worksheets are available to aid understanding. Patients will also be able to access transcripts of their online sessions.

The platform will allow patients to send their therapist a message between sessions, e.g. if they need to rearrange a session, or in relation to an agreed between-session task. Patients will be advised that therapists will address queries about tasks during the next scheduled session.

The expectation is that the first four therapy sessions will take place weekly. Later therapy sessions may be spaced at fortnightly or monthly intervals.

It is possible that patients and therapists may reach an 'agreed end' of therapy in less than nine sessions where clinically appropriate.

In *exceptional* circumstances, it may be clinically appropriate for a therapist to offer a videocall or telephone session instead of an online instant messaging session and/or to offer an additional therapy session (e.g. to enable safeguarding issues to be adequately and sensitively addressed). The justification for additional sessions or face-to-face videocall or telephone contact time will be discussed with the Clinical Supervisor where possible, agreed with the Clinical Principal Investigator (PI), and logged in the clinical notes.

At the end of therapy, patients will be offered the opportunity to download materials (e.g. worksheets, information sheets and therapy session transcripts) from the platform for future use. The patient's GP will be informed that they have completed therapy, if they withdraw from therapy, or are discharged for non-attendance.

Attendance at CBT sessions

Attendance at CBT sessions will be recorded by the patient's therapist on the online therapy platform. The INTERACT therapist will monitor the patient's platform use and therapy attendance. A patient may be discharged from treatment by their therapist if they do not engage with therapy. We have included a qualitative study within our trial to investigate patients' views and experiences of integrated CBT and to identify patients' reasons for completing or not completing therapy.

Patients who are discharged from therapy or who withdraw from therapy will still be invited to complete follow-up assessments and, if sampled, a qualitative interview, with the research team unless they have explicitly indicated that they wish to withdraw from the trial.

Fidelity to the CBT model

All the CBT sessions will be recorded using transcripts of instant messaging sessions or audio-recordings of telephone communication. Transcripts/recordings may be reviewed by the therapist and their clinical supervisor as part of the clinical supervision process.

Patients will provide optional informed consent to their therapy transcripts and/or recordings being accessed for research purposes. Where consent has been provided, recordings will be randomly sampled for evaluation of fidelity to the CBT model by independent CBT expert(s) using a recognised CBT rating scale [49].

During the study there will be no restrictions on the GP's prescribing; for example, if they feel it is necessary to prescribe antidepressant medication.

Platform usage data

Use of the platform (by patients and the research team) will be automatically recorded by the system (for example, when people log into the platform, materials accessed, worksheets edited, sessions attended etc.)

The system will also log detailed interactions with the online CBT materials: which worksheets therapists recommend to, and/or share with patients the most often, whether they modify them, and what modifications are made before sharing. Anonymised platform usage data will be used to understand and describe the delivery of the intervention.

The platform will also record the content of therapy. This includes any editing of session preparation materials (agendas and depression questionnaires), online CBT worksheets and session notes (i.e. information added by therapists and patients) and therapy voice communication/instant messaging transcripts. Access to this information will be restricted to the patient, therapist, their clinical supervisor(s) and, where appropriate, the site clinical lead for the purposes of delivering the therapy. However, patients will be asked to provide additional (optional) written consent for this information to be shared with the wider research team. Where consent is given, this information will be used to understand how the therapy platform (including online CBT worksheets) is used, to inform improvements to the platform's design, to monitor the quality of treatment provided, and look at the skill required to deliver integrated CBT.

Patients, therapists and supervisors will be informed that interactions with the platform will be recorded, and about how this information will be used. All the information collected will be used to help us evaluate the system and integrated approach to delivering CBT. This data will be anonymised and stored for use in future research studies.

Trial comparator — usual care

Participants allocated usual care will continue to receive treatment as usual from their GP. This is not standardised and may include referral to local psychological services provided by IAPT or antidepressant medication, as appropriate. There will be no restrictions on the treatment options that can be offered to this group. However, we will record receipt of psychological therapy received through IAPT or privately as part of the follow-up questionnaires (in addition to other data on treatments and health care usage during the trial).

Participant safety

Trial therapists will be experienced in managing clinical risk, including risk of suicide, self-neglect, harm to self and/or others. Therapists will conduct a risk assessment using questions in line with those used within existing IAPT services and follow the trial SOP with regards to managing risk. Subsequent risk assessments will be triggered by PHQ9 question 9 responses collected as part of therapy, or clinical concern and will be more focused. Therapists must inform the study team of any incident that results in serious harm to the participant or to others and ensure that the incident is fully documented. Should a trial researcher become concerned for the safety of a patient participant (for example, if the participant expresses suicidal ideation or recent self-harm) or be concerned about the safety of others, the researcher will follow the study's safety SOP.

Adverse event reporting

Adverse events (AEs)

Adverse events that might be expected to occur at a higher rate in this group of participants include episodes of self-harm not requiring hospital admission and worsening of depression sufficient to require referral to a clinician. Although these AEs are expected, they will still be reported.

Variations in mood, including worsening of depression that does not lead to self-harm or hospitalisation, are commonly seen during therapy and would not be reported as individual adverse events.

Serious adverse events (SAEs)

Expected SAEs that are a more common occurrence in this study population are self-harm leading to hospitalisation; suicidal attempts leading to hospitalisation; worsening of depression leading to hospitalisation. These expected SAEs will still be reported. Admission to hospital for pre-planned surgery for pre-existing conditions will not be reported as an SAE.

All AEs and SAEs will be assessed by a clinical PI or nominated deputy clinician. We will develop a detailed, SOP for reporting and recording AEs. The Data Monitoring and Ethics Committee (DMEC) will periodically review overall safety data to determine patterns and trends of events, or to identify safety issues, which would not be apparent on an individual case-by-case basis.

Adverse events/reactions will be recorded and reported from the point of consent (baseline assessment) until the 12-month follow-up assessment or point of withdrawal from the study.

Statistical considerations

Outcome measures

Primary outcome The primary outcome will be the score on the Beck Depression Inventory (BDI-II) at 6 months post-randomisation, measured as a continuous variable.

Secondary outcomes Secondary outcomes will include response (at least 50% reduction in depressive symptoms on the BDI-II compared with baseline), remission of symptoms (BDI-II < 10), percentage reduction in depressive symptoms on the BDI-II, depressive symptoms on the PHQ-9, quality of life (EQ-5D-5L and WSAS), and anxiety (GAD-7), all measured at 6 months post-randomisation, together with these outcomes and the continuous score on the BDI-II (primary outcome) measured at 12 months post-randomisation. Data on the PHQ-9 and GAD-7 were also collected as part of the brief telephone follow-ups at 3- and 9-months.

In addition, the EQ-5D-5L will inform the economic evaluation, as will data on the use of primary care consultations and prescribed medication collected from practice medical records. Data on the use of other primary and community care services; secondary care related to mental health, private treatments, use of social services, burden on informal caregivers, personal costs related to mental health and benefits received will also inform the economic evaluation.

Justification of sample size

The NICE depression guidelines group [50] suggested that 0.35SD represents a clinically important difference on our primary outcome, which is approximately 4–5 points on the BDI-II (IPCRESS/CoBalT: standard deviation 12.9/13.9). On this basis, 173 participants in each group give 90% power to detect a difference of 0.35 standard deviations on the BDI-II at a two-sided 5% significance level. Assuming 20% attrition at 6 months, we need to recruit 434 patients.

However, given that referral to IAPT may be part of usual care for the comparator group, it is possible that this may affect the plausibility of the target difference between groups. While it is difficult to determine the impact of this, if the difference to be detected was reduced to 0.30 SD and we achieve a slightly higher follow-up rate (85%) in line with previous trials [8], with a total sample size of 434 patients, we would still have adequate (>80%) power to detect such a difference (see Table 2 below).

We have not inflated our sample size to account for clustering by therapist as there was little evidence of any therapist effects in our previous trials of CBT for depression [2, 8]. The intraclass correlation coefficients for the continuous BDI outcome (adjusted for baseline BDI score) were very small for CoBalT (0.0027) and for IPCRESS, its precise value could not be estimated indicating it was almost zero. Hence, inflating the sample size to account for any potential clustering by therapist would be unduly conservative. Nevertheless, we will use established methods [51] to obtain a fully heteroscedastic model to explore any potential therapist effects in secondary analyses.

Internal pilot

There will be a 9-month internal pilot to ensure that we are able to recruit to the main trial as planned and that participants are engaging with the intervention as expected.

Criteria to judge success of internal pilot

Our progression criteria are that, by the end of the internal pilot phase, we will have achieved:

(1) At least 70% of our anticipated recruitment rate (8 patients per month) in the Bristol site;
(2) At least 70% of those randomised in the Bristol site will have received at least two sessions with a therapist; and
(3) Recruitment will have commenced at the other sites (London and Hull/York). (London and Hull/York will commence set-up four months after the Bristol site.)

Table 2 Sensitivity of sample size calculation to changes in plausible target difference between groups and variations in required power and attrition at 6 months

	Power	80% power		90% power	
	Attrition at 6 months	15%	20%	15%	20%
**Difference to be detected**	0.35 SD	306	326	408	434
	0.30 SD	414	440	554	588

We will employ a traffic-light system to judge the success of our internal pilot. If we achieve at least 70% of our target for recruitment and engagement with the intervention and have commenced recruitment at other sites, then we will continue the study. However, if appropriate, we will explore the reasons for not achieving the target and whether any modifications to recruitment processes are required dependent on the emerging recruitment rate trajectory. If we achieve between 50 and 70% of our recruitment and engagement targets, and/or have not commenced recruitment at other sites, then we will discuss with our Trial Steering Committee whether we should make changes to the recruitment/therapy processes and whether we should continue. If we recruit less than 50% of our recruitment and engagement targets and have not commenced recruitment in other sites, then we would stop the trial. We believe that these progression criteria are realistic based on our experience and account for the fact that recruitment builds over the early months of a trial.

The criteria to judge success were informed by guidance published by Avery et al. [52] and account for the fact that two recruiting sites (London and Hull/York) will have had only a short period to commence recruitment. The second target is based on data from our previous CBT trials [2, 8] in which 9–13% of those randomised to receive the intervention did not attend any sessions and a further 3–7% of participants only attended one session with a therapist, giving an overall percentage of 12–19% attending fewer than two sessions.

Statistical analysis

Analysis and reporting will be in line with CONSORT guidelines [53]. A detailed statistical analysis plan will be prepared and approval sought from the Trial Steering Committee prior to data lock. The analysis plan will be published on the University of Bristol Research Portal. A brief outline of the proposed analysis is presented below.

All analyses will be conducted according to the intention-to-treat principle. Descriptive statistics will be used to ascertain any marked imbalances in demographic or clinical variables at baseline.

Analysis of the primary outcome will use linear regression to compare the groups as randomised, adjusting for stratification/minimisation variables and baseline measurement of the outcome. Analyses of continuous secondary outcomes will be conducted in the same manner as the primary outcome and binary secondary outcomes will be analysed using logistic regression adjusting for stratification/minimisation variables. We will undertake exploratory analyses using regression methods to examine the impact of process measures such as the extent of involvement in CBT/other interventions (including the

use of the INTERACT platform), use of online materials, and therapeutic alliance [43].

In all analyses, we will present regression coefficients (for continuous outcomes) or odds ratios (OR) (for binary outcomes), with 95% confidence intervals and p values. Repeated measures analyses will incorporate outcomes over the 12 months. An interaction between the treatment group and time will formally assess whether treatment effects are sustained or emerge later. We will conduct sensitivity analyses to examine the impact of missing data, baseline imbalances in important prognostic factors, number of treatment sessions attended, potential “therapist effects” and the timing of questionnaire completion. Recognising the inherent biases in per-protocol analyses, a Complier Average Causal Effect (CACE) analysis is planned to estimate the treatment effect among compliers who attended an adequate number of CBT sessions. Sub-group analyses will explore the possibility of differential treatment effects based on the chronicity and severity of depression, and personality difficulties [36].

No planned interim analysis of outcome data will be conducted.

Economic evaluation

Perspectives

An economic evaluation alongside the RCT will estimate the incremental cost-effectiveness of the integrated approach to delivering CBT over and above usual care. The evaluation will be conducted from three perspectives: (1) the National Health Service and personal social services; (2) patients and informal carers; and (3) society.

Outcomes

Resource use data will be collected from three sources: (1) trial records, resources used to deliver the intervention will be recorded throughout the trial; (2) General Practice records—primary care consultations and prescribed medication will be extracted for the 12-month trial period; (3) resource use questionnaire will be administered at 6 and 12 months providing information on the use of hospital and community services; specialist mental health contacts; private treatments; use of social services; burden on informal caregivers; personal costs (e.g. time off work/unpaid activities); and benefits received. Total staff costs to deliver the intervention will be valued assuming mid-point salaries plus salary on-costs. Recognised published sources, such as (Personal Social Services Research Unit Costs of Health and Social Care, National Cost Collection, Prescription Cost Analysis, Labour Market Data by Office for National Statistics, etc.) will be used to apply national average unit costs to service utilisation and construct a cost profile for each patient in the trial.

For outcomes relevant to national policy, cost-utility analysis as recommended by NICE Guidance [54], will examine incremental quality-adjusted life years (QALYs), by collecting information on generic health using the EQ-5D-5L at baseline, 6 and 12 months and calculating the area under the curve [55]. We will use the 5L version of the EQ-5D and the tariff recommended by the latest NICE guidance at the time of analysis. NICE currently recommends the use of the 3L tariff [56], but should any further guidance be subsequently issued we will adhere to that.

Analysis

We will conduct a within-trial incremental cost-effectiveness analysis of delivering the integrated approach to delivering CBT over and above usual care following a health economic analysis plan that will be uploaded to the public domain before data lock. Missing data will be handled primarily using multiple imputation on the assumption of missing at random [57]. The analysis will be undertaken on an intention-to-treat basis. Incremental costs and incremental QALYs will be estimated using linear regression models. Broad and accurate indication of the cost consequences relating to each treatment strategy will provide three alternative scenarios to inform each of the three perspectives. Discounting is not necessary, as the costs and outcomes cover a period of one year only.

Sensitivity analyses will address uncertainties in unit cost estimates or assumptions about missing data. Uncertainty in the cost-effectiveness/utility ratios will be captured by estimating confidence intervals around the net benefit statistic and estimating cost-effectiveness acceptability curves [58].

Nested qualitative study

Method

Semi-structured interviews will be held with the following:

1. people who declined the postal invitation to take part in the trial (‘decliners’), but were willing to be interviewed about their reasons for declining ($n=15$);
2. participants who have completed their (6-month) primary outcome measures for the trial ($n=30$);
3. participants who have completed their (12-month) outcome measures for the trial (‘exit interviews’, $n=30$);
4. trial CBT therapists ($n=9$) and their clinical supervisors ($n=3$).

All interviews will be semi-structured and will be audio-recorded and transcribed verbatim. Topic guides will be used to ensure consistency. The therapist and supervisor topic guides will include some of the areas covered in the trial participants' topic guide, so that we can triangulate participants', therapists' and supervisors' accounts to identify similarities and differences in their views and experiences.

For each set of interviews held with patients, i.e. with decliners and trial participants, individuals will be purposefully sampled to ensure maximum variation in relation to specific variables to ensure we capture diverse views and experiences (Table 3).

Exit interviewees will be sampled from those who have indicated they are willing to be interviewed about their responses to the 12-month exit questionnaire (e.g. regarding their reasons for taking part in the study and their experience of being in the trial).

Immediately prior to interview, the qualitative researcher will remind the individual of the purpose of the interview, answer any questions they may have and obtain written (or verbal if over the telephone or videocall) consent.

We will interview trial therapists and their clinical supervisors once the therapists have completed delivering treatment to most or all of their INTERACT clients. Therapists and supervisors will also be asked to complete a brief demographic questionnaire, so that we can describe the sample when analysing the data and reporting findings.

Analysis

Data collection and analysis will proceed in parallel, so that analytical insights from earlier interviews can shape later data collection. The patient, therapist, and supervisor interviews will be analysed thematically [60].

Transcripts will be read and re-read by members of the research team to familiarise themselves with the data, identify themes and develop a coding frame. Coding frames will be developed for each interview set, e.g., participants, therapists and supervisors, decliners and participants exiting the trial. Once a coding frame has been agreed upon, transcripts will be imported into the software package NVivo to allow electronic coding and data retrieval. Following this, data pertaining to each code will be summarised in tables using an approach based on Framework analysis [61]. This will enable comparisons to be made within and across the interviews, to identify thematic patterns and deviant cases, highlighting the views participants hold toward specific issues, e.g. the value of the initial face-to-face videocall and the use of worksheets between sessions.

Data management

Electronically-captured baseline data (e.g. CIS-R data) will be imported into the trial database by the research team. If participants complete online questionnaires, their responses will be automatically recorded in the trial database. Paper questionnaire data will be manually entered onto the study database in electronic form by the researcher.

Table 3 Summary of planned qualitative interviews

	Sample size	Modality	Interview length (minutes)	Sampling
Decliners	15 [a]	Telephone	≤ 20	By age and gender from those indicating that: they did not want to take part in a research study; did not want CBT; felt they wouldn't benefit from CBT; or didn't want to receive online therapy
Trial participants (after primary outcome completion)	30 [a] (20 intervention and 10 usual care)	In person, telephone or videocall	≤ 60	By site, trial arm, age, gender, socio-economic background, depression severity and for individuals in the intervention arm, treatment response, adherence to treatment and therapeutic alliance score
Trial participants (after trial completion) 'Exit interviews'	30 [a]	Telephone or videocall	≤ 20	By site, trial arm, age, gender, ethnicity, depression duration, depression score (baseline and at follow-ups) and for the intervention arm—therapist, number of sessions received and therapy adherence (i.e. whether they completed or withdrew from therapy)
Trial therapists	9	In person, telephone or videocall	≤ 60	All
Therapists' clinical supervisors	3	In person, telephone or videocall	≤ 60	All

[a] The final number will be guided by information power [59]

Trial databases will incorporate data entry and validation rules to reduce data entry errors, and management functions to facilitate auditing and data quality assurance.

Most of the therapy data will be recorded directly on the INTERACT platform. Other therapy data (e.g. initial clinical assessment and notes regarding the management of risk) will be logged on electronic forms.

The database and randomisation system will be designed to protect participant information in line with the data protection regulations. Participants will be identified only by a patient ID number on questionnaires, interview transcripts and the randomisation system. Trial documents will be stored securely and made accessible only to trial staff and authorised personnel. Data will be anonymised as soon as it is practical to do so.

All identifiable essential data will be stored for 10 years, and anonymised digital data will be stored indefinitely. The Chief Investigator is the data custodian.

Monitoring, audit and inspection

Trial monitoring is undertaken by University Hospitals Bristol and Weston (UHBW) on behalf of the Sponsor A monitoring plan will be developed to include self-monitoring at sites (e.g. checking of site file documentation, safety reporting, and consent form reviews) and database checking and source data verification checks.

Ethical and regulatory considerations

The trial will be conducted in compliance with the principles of the Declaration of Helsinki (1996), the principles of Good Clinical Practice and in accordance with all applicable regulatory requirements. This protocol and related documents will be submitted to a Research Ethics Committee and the Health Research Authority (HRA) for review. Any subsequent amendments to the study protocol or related documents will be submitted to the appropriate regulatory bodies for approval in accordance with guidelines. Participating sites will be notified of amendments so they can put arrangements in place to implement the amendment if necessary and confirm their support for the study as amended.

Public and Patient Involvement (PPI)

Patients, service users and members of the public have been involved in the design and management of this research and will also be involved in dissemination activities. We conducted substantial pilot work to gather stake-holder feedback on the trial intervention prior to undertaking this trial [18-21].

The INTERACT programme includes PPI members on its Management Group and Independent Steering Committee. PL a service user representative co-applicant, and member of the management group responsible for developing the intervention. PPI members of these groups have also provided feedback on patient information for trial participants.

Dissemination policy

Dissemination will be in accordance with the INTERACT programme dissemination policy. Trial results will be published in leading international peer-reviewed journals and presented at national and international conferences. A detailed final report of the INTERACT programme will be published by the funder, NIHR. We will disseminate findings to all relevant stakeholder groups including participating GP practices and will send a summary of the trial results to those trial participants who have indicated they would like to receive this following publication of the study findings.

Discussion

The INTERACT trial will be the first large multi-centre trial to investigate the clinical and cost-effectiveness of an integrated approach to delivering CBT in the UK over 12 months. The nested qualitative study provides insight into patients', therapists' and supervisors' views and experiences of this approach to treating depression. Trial participants will be recruited from a wide range of primary care practices across the three study centres, based in urban and rural, affluent and deprived communities across the UK, thus maximising the generalisability of trial findings. Therapists will have a comparable experience to those working as high-intensity therapists within IAPT services, ensuring that the results of the trial apply to standard clinical settings in the UK.

INTERACT is a trial that is highly relevant to patients and the NHS, particularly in the context of increased levels of depression and anxiety in the community following the COVID-19 pandemic. The need for online delivery of psychological therapy is now widely recognised, yet there is very little evidence of the effectiveness of high-intensity online interventions. The results of the INTERACT trial will provide a robust estimate of the clinical and cost-effectiveness of this novel approach to delivering CBT and hence the evidence base for the treatment of depression in UK primary care.

Trial status

The current protocol is version 2.0 (24/03/2023). The study opened to participant recruitment on 9/12/2020. It is expected that recruitment will end in about May 2023 and data collection will be completed in June 2024.

Appendix

Composition and roles of the Trial Steering Committee, Data Monitoring Committee and Trial Management Group.

Trial Steering Committee (TSC)

The TSC is an independent, multidisciplinary group chaired by André Tylee (Professor of Primary Care Mental Health) until July 2021, by Sandra Eldridge (Professor of Biostatistics) until October 2021, and thereafter by Ed Watkins (Professor of Experimental and Applied Clinical Psychology). Other members are Shaun Lawson (Professor of Social Computing), Philip Pallmann (Deputy Director Research Design and Conduct Service and Research Fellow), Michael Moore (Professor of Primary Health Care Research), Marta Sawinska (patient representative), and Mark Tucker (patient representative). Their role is to provide independent oversight, progress monitoring, and expert advice during the conduct of the trial. TSC meetings are also attended by NW, DK, TP, SM and DT from the INTERACT team. Meetings take place at least annually and more frequently if judged necessary.

This group has previously acted as the Programme Steering Committee for the INTERACT programme of research.

Data Monitoring and Ethics Committee (DMEC)

The DMEC is an independent, multidisciplinary group whose role is to safeguard the interests of trial participants, monitor the safety and efficacy of the intervention, and monitor the overall conduct of the study. The DMEC will advise the TSC on whether there are any ethical or safety reasons why the trial should not continue. DMEC meetings will be held at least annually but more frequently if necessary. The DMEC comprises David Kingdon (DMEC Chair; Emeritus Professor of Mental Health Care Delivery), Chris Burton (Professor of Primary Medical Care), and Nikki Totton (Medical Statistician). A DMEC Charter outlines the role and responsibilities of the DMEC, and any competing interests.

Trial Management Group (TMG)

The Trial Management Group (TMG) will be led by the INTERACT programme leads, DK and NW. It will comprise all investigators involved in the trial, the trial manager, research and administrative staff, with input from patient/public representatives. Members of the TMG contribute to the trial in the following ways: trial design, trial centre recruitment and trial conduct, trial management, trial logistics and cost management, CBT expertise, economic evaluation, trial methods, statistical data analysis, and publication.

The TMG will meet approximately monthly to oversee the day-to-day management of the trial. The TMG will be provided with detailed information by site staff regarding trial progress. Most meetings will be by teleconference/videocall.

Abbreviations

AE	Adverse event
AUDIT-PC	Alcohol Use Disorders Identification Test Primary Care
BABCP	British Association for Behavioural & Cognitive Psychotherapies
BDI-II	Beck Depression Inventory-II
BFI	Big Five Personality Inventory (BFI)
BTC	Bristol Trials Centre
cCBT	Computerised cognitive behavioural therapy
CBT	Cognitive behavioural therapy
CI	Chief Investigator
CRF	Case report form
CRN	Clinical Research Network
DMEC	Data Monitoring and Ethics Committee
GAD-7	Generalised Anxiety Disorder-7
GCP	Good Clinical Practice
GDPR	General Data Protection Regulations
HRA	Health Research Authority
IAPT	Improving Access to Psychological Therapies
ICD-10	International Statistical Classification of Diseases and Related Health Problems 10th Revision
ISRCTN	International Standard Randomised Controlled Trials Number
MRI	Magnetic resonance imaging
NICE	National Institute for Health and Care Excellence
NIHR	National Institute for Health Research
PC-PTSD-5	Primary Care Post-Traumatic Stress Screen-5
PHQ-9	Patient Health Questionnaire-9
PI	Principal Investigator
PIC	Participant Identification Centre
PPI	Public and Patient Involvement
QALY	Quality-adjusted life year
RCT	Randomised controlled trial
REC	Research Ethics Committee
SAE	Serious adverse event
SAPAS	Structured Assessment of Personality Abbreviated Scale
SOP	Standard operating procedure
TMG	Trial Management Group
TSC	Trial Steering Committee
UCL	University College London
UHBW	University Hospitals Bristol and Weston NHS Foundation Trust
WAI-SR	Working Alliance Inventory Short Revised
WAI-SRT	Working Alliance Inventory Short Revised – Therapist
WSAS	Work and Social Adjustment Scale

Supplementary Information

The online version contains supplementary material available at https://doi.org/10.1186/s13063-023-07396-9.

Additional file 1. SPIRIT Checklist.

Acknowledgements

This study was designed and delivered in collaboration with the Bristol Trials Centre, a UKCRC-registered Clinical Trials Unit. We are grateful to the INTERACT therapists and clinical supervisors who provided invaluable advice and feedback relating to the INTERACT platform, and to colleagues from the local Clinical Research Networks who supported recruitment activities. We are also grateful to a number of colleagues who are involved with the INTERACT study as co-applicants but who have not participated in drafting this manuscript: Rachel Churchill, David Coyle, Una Macleod, Nicky Welton and Catherine

Wevill. Study data were collected and managed using REDCAP [62], hosted at the University of Bristol.

Authors' contributions

AB contributed to the development of the integrated CBT intervention and provided clinical supervision for intervention delivery. BCFC contributed to trial coordination, data collection and data management at the UCL site. BJ contributed to trial coordination, data collection and data management at the UCL site. DT contributed to study concept and design; study management; data management and data collection; drafted the original protocol and the first draft of the manuscript. DK and NW are the INTERACT study co-leads. They conceived and designed the study and trial intervention; they led the grant application and management of the study. FF was responsible for the data collection and analysis of the nested qualitative study. GL contributed to study concept and design; managed the UCL site; contributed to the funding application. IN contributed to study concept and design; contributed to the funding application. JL contributed to the design and management of the health economic evaluation. JSH contributed to trial coordination, data collection and data management at the UCL site. KT contributed to study concept and design; was responsible for the design and management of the nested qualitative study and qualitative analysis; contributed to funding application. LT contributed to study concept and design; study management; data management and data collection. MXL contributed to trial coordination, data collection and data management at the UCL site. PL contributed to study concept and design and the funding application; provided ongoing PPI expertise and guidance throughout the study. RS and CW contributed to study concept and design; the design of the intervention; and the funding application. SB contributed to trial coordination, data collection and data management at the University of York site. SG contributed to study concept and design; managed the University of York site; and contributed to the funding application. SJM contributed to statistical analysis design; and data management design. SP contributed to study concept and design; was responsible for the design and management of the health economic evaluation; and contributed to funding application. TJP contributed to study concept and design; statistical analysis design; and the funding application. All authors read, contributed to and approved the final manuscript.

Funding

This report is independent research funded by the National Institute for Health and Care Research (Programme Grants for Applied Research, Integrated therapist and online CBT for depression in primary care, RP-PG-0514–20012). The views expressed in this publication are those of the author(s) and not necessarily those of the NHS, the NIHR, the Department of Health or the Sponsor. The funder and Sponsor have no role in the study design, data collection, data analysis, interpretation of data, or manuscript writing. Dissemination of results will include the publication of a detailed report, written by the INTERACT study team and published by the NIHR.
Supplementary funding was provided by the NIHR School for Primary Care Research (SPCR—2014–10043 Award: L)."
This study was also supported by the NIHR Biomedical Research Centre at the University Hospitals Bristol and Weston NHS Foundation Trust and the University of Bristol (grant number BRC-1215–20011). The NIHR Bristol Biomedical Research Centre funded the fMRI add on study.
Excess Treatment Costs have been awarded to cover the cost of delivering the intervention. The NIHR Clinical Research Network has provided Service Support Cost payments to participating General Practices and local research staff to support INTERACT.

Availability of data and materials

Data from the INTERACT trial will be lodged with the University of Bristol Research Data Repository and assigned a unique DOI. Access to INTERACT data for non-commercial research will be through a system of managed access (University of Bristol 'controlled access'). The final decision to release data will be taken by the University of Bristol's Data Access Committee. There may be a charge for preparing the dataset. Requests for data any other purposes must be negotiated separately.

Declarations

Ethics approval and consent to participate

This study was approved by the South West – Frenchay Research Ethics Committee (REC reference: 19/SW/0038) and Health Research Authority (HRA). Informed consent will be obtained from all participants prior to participation in the trial.

Consent for publication

Not applicable. No participant data has been included in this protocol.

Competing interests

CW is the author of written and online CBT self-help resources and is the Director of a company which commercialises these resources. The other authors have no conflict of interests with respect to this protocol.

Author details

[1]Bristol Medical School, Centre for Academic Mental Health, Population Health Sciences, University of Bristol, Oakfield House, Oakfield Grove, Bristol BS8 2BN, UK. [2]ARRC 208, Department of Health Sciences, University of York, York YO10 5DF, UK. [3]UCL Division of Psychiatry, Maple House, 149 Tottenham Court Road, London WIT 7BN, UK. [4]Mental Health & Addiction Research Group, University of York & Hull York Medical School, York, UK. [5]Public and Patient Involvement Representative, London, UK. [6]Department of Health Sciences, Seebohm Rowntree Building, University of York, Heslington, York YO10 5DD, UK. [7]Bristol Medical School, Population Health Sciences, University of Bristol, Bristol, UK. [8]Department of Primary Care & Population Health, University College London, Royal Free Site, Rowland Hill Street, London NW3, UK. [9]Bristol Dental School, University of Bristol, Lower Maudlin Street, Bristol BS21 2LY, UK. [10]Great Ormond Street Institute of Child Health London, University College London, London WC1N 1EH, UK. [11]Centre for Academic Primary Care, Bristol Medical School, Population Health Sciences, University of Bristol, Canynge Hall, 39 Whatley Road, Bristol BS8 2PS, UK. [12]Clarice Pears Building, University of Glasgow, 90 Byres Road, Glasgow G12 8TA, UK. [13]Five Areas Ltd, 1 Aurora Avenue, Clydebank G81 1BF, UK.

Received: 30 March 2023 Accepted: 19 May 2023
Published online: 20 June 2023

References

1. Cuijpers P, Cristea I, Karyotaki R, et al. How effective are cognitive behavior therapies for major depression and anxiety disorders? A meta-analytic update of the evidence. World Psychiatry. 2016;15:245–58.
2. Wiles N, Thomas L, Abel A, et al. Cognitive behavioural therapy as an adjunct to pharmacotherapy for primary care based patients with treatment resistant depression: results of the CoBalT randomised controlled trial. Lancet. 2013;381:375–84.
3. NHS England. NHS Talking Therapies, for anxiety and depression. https://www.england.nhs.uk/mental-health/adults/nhs-talking-therapies. Accessed 16 Mar 2023.
4. Department of Health. Talking therapies: a four-year plan of action. 2011. https://www.gov.uk/government/publications/talking-therapies-a-4-year-plan-of-action. Accessed 2015.
5. Radhakrishnan M, Hammond G, Jones P, et al. Cost of Improving Access to Psychological Therapies (IAPT) programme: an analysis of cost of session, treatment and recovery in selected primary care trusts in the East of England region. Behav Res Ther. 2013;51:37–45. https://doi.org/10.1016/j.brat.2012.10.001.
6. Richards D, Richardson T. Computer-based psychological treatments for depression: a systematic review and meta-analysis. Clin Psychol Rev. 2012;32:392–342.
7. Helgadottir F, Menzies R, Onslow M, et al. Online CBT I: Bridging the gap between Eliza and modern online CBT treatment packages. Behav Chang. 2009;26:245–53.
8. Kessler D, Lewis G, Kaur S, et al. Therapist-delivered internet psychotherapy for depression in primary care: a randomised controlled trial. Lancet. 2009;374:628–34.

9. Hollinghurst S, Peters T, Kaur S, et al. Cost-effectiveness of therapist-delivered online cognitive behavioural therapy for depression: randomised controlled trial. Br J Psychiatry. 2010;197:297–304.
10. Hiley C. UK mobile phone statistics, 2023. https://www.uswitch.com/mobiles/studies/mobile-statistics. Accessed 16 Mar 2023.
11. Barnes M, Sherlock S, Thomas L, et al. No pain, no gain: depressed clients' experiences of cognitive behavioural therapy. Br J Clin Psychol. 2013;52:347–64.
12. Westra H, Dozois D, Marchus M. Expectancy, homework compliance, and initial change in cognitive-behavioral therapy for anxiety. J Consult Clin Psychol. 2007;75:363–73.
13. Mausbach M, Moore R, Roesch S, et al. The relationship between homework compliance and therapy outcome: an updated meta-analysis. Cognit Ther Res. 2010;34:429–38.
14. Wu Q, Li J, Parrott S, et al. Cost-effectiveness of different formats for delivery of cognitive behavioural therapy for depression: a systematic review based economic model. Value Health. 2020;23:1662–70.
15. López-López JA, Davies SR, Caldwell DM, et al. The process and delivery of CBT for depression in adults: a systematic review and network meta-analysis. Psychol Med. 2019;49:1937–47.
16. Stawarz K, Preist C, Coyle D. Use of smartphone apps, social media, and web-based resources to support mental health and well-being: online survey. JMIR Ment Health. 2019;6: e12546.
17. Stawarz K, Preist C, Tallon D, et al. User experience of CBT apps for depression: an analysis of app functionality and user reviews. J Med Internet Res. 2018;20: e10120.
18. Taylor A, Tallon D, Kessler D, et al. An expert consensus on the most effective components of cognitive behavioural therapy for adults with depression: a modified Delphi study. Cogn Behav Ther. 2020;49:242–55.
19. Tallon D, McClay C, Kessler D, et al. Materials used to support cognitive behavioural therapy for depression: a survey of therapists' clinical practice and views. Cogn Behav Ther. 2019;48:463–81.
20. Stawarz K, Preist C, Tallon D, et al. Design considerations for the integrated delivery of cognitive behavioral therapy for depression: user-centered design study. JMIR Ment Health. 2020;7: e15972.
21. Stawarz K, Preist C, Tallon D, et al. Integrating the digital and the traditional to deliver therapy for depression: lessons from a pragmatic study. Proceedings of the 2020 CHI Conference on Human Factors in Computing Systems; 2020 Apr 25–30; Honolulu, HI, USA. Association for Computer Machinery; 2020. Available from: https://dl.acm.org/doi/pdf/10.1145/3313831.3376510.
22. World Health Organization. International Clinical Trials Registry Platform (ICTRP) Search Portal. https://trialsearch.who.int. Accessed 2015.
23. E-COMPARED. E-COMPARED European Comparative Effectiveness Research on Internet-based Depression Treatment. https://www.e-compared.eu. Accessed 26 Feb 2023.
24. NHS England. NHS Mental Health Implementation Plan 2019/20 - 2023/24. 2019. https://www.longtermplan.nhs.uk/publication/nhs-mental-health-implementation-plan-2019-20-2023-24. Accessed 2023.
25. Chan A-W, Tetzlaff JM, Gøtzsche PC, et al. SPIRIT 2013 explanation and elaboration: guidance for protocols of clinical trials. BMJ. 2013;346: e7586.
26. Lewis G. Assessing psychiatric disorder with a human interviewer or a computer. J Epidemiol Community Health. 1994;48:207–10.
27. Lewis G, Pelosi A, Araya R, et al. Measuring psychiatric disorder in the community: a standardized assessment for use by lay interviewers. Psychol Med. 1992;22:465–86.
28. National Institute for Health and Care Excellence (NICE). Depression in adults: treatment and management. NICE guideline NG222, 2022. Available from: https://www.nice.org.uk/guidance/ng222
29. Beck A, Steer R, Brown G. Manual for the Beck Depression Inventory-II. San Antonio, TX: Psychological Corporation; 1996.
30. NHS England. GP Patient Survey. http://www.gp-patient.co.uk/about. Accessed 10 Jan 2019.
31. Kroenke K, Spitzer R, Williams J. The PHQ-9: validity of a brief depression severity measure. J Gen Intern Med. 2001;16:606–13.
32. Spitzer R, Kroenke K, Williams J, et al. A brief measure for assessing generalized anxiety disorder: the GAD-7. Arch Intern Med. 2006;166:1092–7.
33. Herdman M, Gudex C, Lloyd A, et al. Development and preliminary testing of the new five-level version of EQ-5D (EQ-5D-5L). Qual Life Res. 2011;20:1727–36.
34. Mundt J, Marks I, Shear K, et al. The Work and Social Adjustment Scale: A simple measure of impairment in functioning. Br J Psychiatry. 2002;180:461–4.
35. John O, Donahue E, Kentle R. The Big Five Inventory - Versions 4a and 5. Berkeley, CA: University of California, Berkeley, Institute of Personality and Social Research; 1991.
36. Moran P, Leese M, Tennyson L, et al. Standardised assessment of personality - abbreviated scales (SAPAS): preliminary validation of a brief screen for personality disorder. Br J Psychiatry. 2003;183:228–32.
37. Prins A, Bovin M, Kimerling R, et al. The Primary Care PTSD Screen for DSM-5 (PC-PTSD-5). [Measurement instrument]. 2015. https://www.ptsd.va.gov/professional/assessment/screens/pc-ptsd.asp. Accessed 2015.
38. Saunders J, Aasland O, Babor T, et al. Development of the Alcohol Use Disorders Identification Test (AUDIT): WHO collaborative project on early detection of persons with harmful alcohol consumption–II. Addiction. 1993;88:791–804.
39. Thomas L, Abel A, Ridgway N, et al. Cognitive behavioural therapy as an adjunct to pharmacotherapy for treatment resistant depression in primary care: the CoBalT protocol. Contemp Clin Trials. 2012;33:312–9.
40. Brown S, Thorpe H, Hawkins K, et al. Minimization - reducing predictability for multi-centre trials whilst retaining balance within centre. Stat Med. 2005;24:3715–27.
41. Chalder M, Wiles N, Campbell J, et al. Facilitated physical activity as a treatment for depressed adults: randomised controlled trial. BMJ. 2012;344: e2758.
42. Hamilton M. A rating scale for depression. J Neurol Neurosurg Psychiatry. 1960;23:56–62.
43. Horvath A, Greenberg L. Development and validation of the working alliance inventory. J Counsel Psychol. 1989;36:223–33.
44. Beck A, Rush A, Shaw B, et al. Cognitive Therapy of Depression. NY: Guildford Press; 1979.
45. Beck J. Cognitive therapy: Basics and beyond. New York: Guilford Press; 1995.
46. Beck J. Cognitive Behaviour Therapy: basics and beyond. 2nd ed. New York: Guildford Press; 2011.
47. Roth A and Pilling S. The competences required to deliver effective cognitive and behavioural therapy for people with depression and with anxiety disorders. Department of Health; 2007. https://www.ucl.ac.uk/clinical-psychology//CORE/CBT_Competences/CBT_Competence_List.pdf. Accessed 2015.
48. Roth A and Pilling S. The map of CBT competences for depression and anxiety disorders. 2015. https://www.ucl.ac.uk/clinical-psychology/competency-maps/cbt-map.html. Accessed 26 Feb 2023.
49. Blackburn I-M, James IA, Milne DL, et al. The revised cognitive therapy scale (CTS-R): psychometric properties. Behavioural and Cognitive psychotherapy. 2001;29:431-46.
50. National Institute for Clinical Excellence. Depression: Management of Depression in Primary and Secondary Care. London: England; 2004.
51. Roberts C, Roberts S. Design and analysis of clinical trials with clustering effects due to treatment. Clin Trials. 2005;2:152–62.
52. Avery K, Williamson P, Gamble C, et al. Informing efficient randomised controlled trials: exploration of challenges in developing progression criteria for internal pilot studies. BMJ Open. 2017;7: e013537.
53. Schulz K, Altman D, Moher D, et al. CONSORT 2010 statement: updated guidelines for reporting parallel group randomized trials. Ann Intern Med. 2010;152:716–32.
54. National Institute for Health and Care Excellence (NICE). Guide to the methods of technology appraisal 2013: process and methods. PMG9. 2013. Available from: https://www.nice.org.uk/process/pmg9/
55. Richardson G, Manca A. Calculation of quality adjusted life years in the published literature: a review of methodology and transparency. Health Econ. 2004;14:1203.
56. National Institute for Health and Care Excellence (NICE). Position statement on use of the EQ-5D-5L valuation set for England (updated October 2019). 2019. https://www.nice.org.uk/about/what-we-do/our-programmes/nice-guidance/technology-appraisal-guidance/eq-5d-5l. Accessed 24 Mar 2023.
57. Rubin D. Statistical matching using file concatenation with adjusted weights and multiple imputations. J Bus Econ Stat. 1986;4:87–94.
58. Fenwick ECK, Sculpher M. Representing uncertainty: the role of cost-effectiveness acceptability curves. Health Econ. 2001;10:779–87.

59. Malterud K, Siersma VD, Guassora AD. Sample size in qualitative interview studies: guided by information power. Qual Health Res. 2016;26:1753–60.
60. Braun V, Clarke V. Using thematic analysis in psychology. Qual Res Psychol. 2006;3:77–101.
61. Ritchie J, Spencer L. Qualitative data analysis for applied policy research. In: Bryman A, Burgess R, editors. Analysing qualitative data. London: Routledge; 1994. p. 173–94.
62. Harris P, Taylor R, Theilke R, et al. Research electronic data capture (REDCap) - a metadata-driven methodology and workflow process for providing translational research informatics support. J Biomed Inform. 2009;42:377–81.

Publisher's Note

Springer Nature remains neutral with regard to jurisdictional claims in published maps and institutional affiliations.

Ready to submit your research? Choose BMC and benefit from:

- fast, convenient online submission
- thorough peer review by experienced researchers in your field
- rapid publication on acceptance
- support for research data, including large and complex data types
- gold Open Access which fosters wider collaboration and increased citations
- maximum visibility for your research: over 100M website views per year

At BMC, research is always in progress.

Learn more biomedcentral.com/submissions

BMC
